# Evolution from Covalent to Self-Assembled PAMAM-Based Dendrimers as Nanovectors for siRNA Delivery in Cancer by Coupled In Silico-Experimental Studies. Part I: Covalent siRNA Nanocarriers

**DOI:** 10.3390/pharmaceutics11070351

**Published:** 2019-07-18

**Authors:** Domenico Marson, Erik Laurini, Suzana Aulic, Maurizio Fermeglia, Sabrina Pricl

**Affiliations:** Molecular Biology and Nanotechnology Laboratory (MolBNL@UniTS), Department of Engineering and Architecture, University of Trieste, 34127 Trieste, Italy

**Keywords:** RNAi therapeutics, siRNA delivery, covalent dendrimers, PAMAM dendrimers, nanovectors, gene silencing

## Abstract

Small interfering RNAs (siRNAs) represent a new approach towards the inhibition of gene expression; as such, they have rapidly emerged as promising therapeutics for a plethora of important human pathologies including cancer, cardiovascular diseases, and other disorders of a genetic etiology. However, the clinical translation of RNA interference (RNAi) requires safe and efficient vectors for siRNA delivery into cells. Dendrimers are attractive nanovectors to serve this purpose, as they present a unique, well-defined architecture and exhibit cooperative and multivalent effects at the nanoscale. This short review presents a brief introduction to RNAi-based therapeutics, the advantages offered by dendrimers as siRNA nanocarriers, and the remarkable results we achieved with bio-inspired, structurally flexible covalent dendrimers. In the companion paper, we next report our recent efforts in designing, characterizing and testing a series of self-assembled amphiphilic dendrimers and their related structural alterations to achieve unprecedented efficient siRNA delivery both in vitro and in vivo.

## 1. RNA Interference and Challenges in Small Interference RNA Therapeutics

Discovered in 1986 by the Nobel laureates Fire and Mello [1], RNA interference (RNAi)—also known as post-transcriptional gene silencing (PTGS)—is a compendium of mechanisms involving small RNAs that regulate the expression of genes in a variety of eukaryotic organisms. In simple terms, the RNAi process implies the cleavage of endogenous long double-stranded RNAs into short ribonucleic acid sequences (the so called small interfering RNAs or siRNAs, usually 21–23 bases long) by the action of the Dicer endonuclease [2]. Upon incorporation into the multiprotein RNA-induced silencing complex (RISC), the double helical siRNAs are unwound into two strands: The sense (or passenger) strand is discarded, and the antisense (or guide) strand is paired to a complementary mRNA sequence via the RISC complex. Upon binding, the targeted mRNA is in turn degraded by a RISC subunit (known as Argonaute 2) endowed with endonuclease activity. This last step ultimately results in the prevention of mRNA translation into the corresponding protein or, in other words, in gene silencing. Finally, once the target mRNA degradation is accomplished, the RISC complex can be recycled to digest other targets on the mRNA, greatly improving inhibition efficiency [3].

In the post-genomic era, siRNAs of a synthetic nature can be designed and synthesized under good manufacturing practice (GMP) to target complementary regions on any gene of a known sequence to achieve its downregulation for curative purposes [4,5,6]. However, key to the therapeutic utility of these RNAi triggers is the ability to introduce them into their target cells of the human body. Indeed, naked siRNAs are not amenable to therapeutic administration. For instance, when a siRNA is administered intravenously, it is readily digested by nucleases and largely cleared from the kidney glomeruli before reaching the diseased organs. Moreover, the negative charge and large size of a naked siRNA make it difficult to pass through the plasma membrane of a target cell. Even if the siRNA molecules could reach the target tissue and be taken up by target cells, they must avoid degradation in lysosomes via endosomal escape, a process in which the efficiency of these nucleic acid fragments is notoriously low. As a consequence, one of the major challenges in successful RNAi therapeutics is the discovery of safe, efficient and effective siRNA delivery vectors. Such delivery vehicles must at least protect each siRNA from nucleases in the serum or extracellular media, enhance siRNA transport across the cell membrane, and guide the siRNA to its proper location through interactions with the intracellular trafficking machinery. Ideally, both viral and non-viral (nano)vectors can deliver siRNA into cells [7], although, despite impressive transfection efficiency, the use of the former is limited by safety concerns, including genotoxic and immunogenicity-mediated adverse events [8]. On the contrary, non-viral delivery systems have great potential for the safer delivery of siRNA therapeutics, although so far their performance in transfection efficiency has not reached the level requested for full clinical exploitation [7,9,10,11].

## 2. Role of Dendrimers as siRNA Nanocarriers

**Figure 1:**
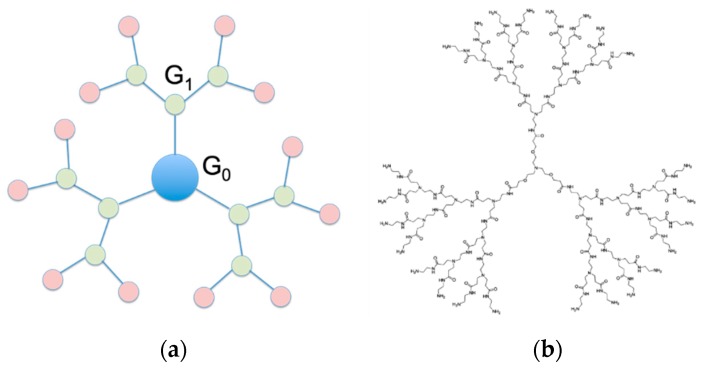
(**a**) Cartoon representation of a dendrimer structure highlighting its three, distinct structural motifs: The core (in light blue), the branching units forming the different G generations (in light green), and the terminal groups (in light pink). According to a consolidate dendrimer nomenclature, the core constitutes Generation 0 (G_0_). Therefore, the subsequent Generations G_1_, G_2_, … G_n_ refer to the corresponding level of branching, as annotated in panel (**a**). (**b**) Molecular structure of the triethanolamine (TEA)-core poly(amidoamine) dendrimers. For clarity, in panel (**b**), only a dendrimer of Generation 4 (G_4_) is shown.

In the variegated scenario of non-viral (nano)materials for siRNA delivery, dendrimers have quickly grown as a family of synthetic nano-sized, radially symmetric molecules with fine-defined, homogeneous and monodisperse composition endowed with enormous potential as gene therapy nanovectors [12,13,14]. Structurally, dendrimers are constituted by three distinct domains (Figure 1a): (1) A fundamental atom or, most frequently, a group of atoms defined as the core; (2) the branching units, which, emanating from the core through diverse chemical reactions, allow the dendrimeric molecule to grow in geometrically organized radial layers known as generations (G); and (3) an exponentially increasing number of peripheral surface groups which constitute a multivalent nanoscale array and can therefore form high-affinity interactions with a variety of biological targets [15].

From the synthetic viewpoint, both divergent and convergent pathways (or combinations of thereof) can be adopted to prepare dendrimers with different generations with precisely defined, regular structures [16,17]. To date, more than fifty families of dendrimers each with unique chemistry and properties have been produced and are under investigation in a diversity of different biomedical applications [18]. Among these, poly(amidoamine) (aka PAMAM) dendrimers undoubtedly constitute the molecules most widely explored as nanocarriers for both drug and gene delivery [13,19,20,21]. In the specific field of nucleic acid delivery and release, this popularity can be ascribed to several beneficial features of PAMAMs, including (i) the chemical nature of their terminal groups, which, being primary amines, are fully protonated at the physiological pH of 7.4. This entails extremely favorable electrostatic (Coulombic) interactions of these dendrimers with the negatively charged nucleic acid fragments and the subsequent mutual condensation into nanoscopic particles, often called dendriplexes; and (ii) the presence of tertiary amines within the dendritic branched structure which, becoming protonated at lower pH values pertaining to endosomes and lysosomes, mediate the osmotic swelling and subsequent disruption of the membranes of these vesicles, ultimately promoting the intracellular release of the siRNA cargo. This mechanism, known as the proton-sponge hypothesis, relies on the assumption (under debate; [22]) that the unprotonated amines of PAMAMs can absorb protons as they are pumped into the lysosome/endosome, resulting in more protons being pumped in, thus leading to an increased influx of Cl^−^ ions and water. A combination of the osmotic swelling and a swelling of the dendrimers themselves because of the repulsion between protonated amine groups causes the rupture of the lysosomal/endosomal membranes, resulting in the subsequent release of its contents into the cytoplasm [23].

## 3. Structurally Flexible PAMAM Dendrimers for Safe, Efficient and Effective siRNA Delivery

Despite the wealth of studies dating back to the early 90s yielding highly promising results for PAMAM-based dendrimers as DNA nanovectors [24,25,26,27,28], only the last 10 years have witnessed systematic investigations of this class of molecules in siRNA delivery (see Table S1 in Supplementary Materials) [19,29,30,31,32]. In this arena, successive rounds of structure optimization led us to the design of PAMAM dendrimers with a triethanolamine (TEA) core (Figure 1b) [33]. The rationale behind the conception and synthesis of this new dendrimer family was that the TEA-core molecules, having the branching units starting away from the central amine with a distance of 10 successive bonds, should feature an extended core. As such, they were expected to be less congested in space with respect to the prototypical NH_3_-core PAMAM dendrimers, in which the branches sprout directly from the small ammonia focal point. As a consequence, the TEA-core dendrimers—with less densely packed branches and terminal units—should be endowed with an enhanced flexibility of their arms and, as such, should perform better as siRNA nanocarriers than their NH_3_-core counterparts. In essence, the hypothesis of a greater flexibility translating into the more effective enwrapping of the nucleic acid fragment was inspired by the behavior of histones, whose structure dynamics allows for conformational changes related to DNA binding (required for post-translational modification) and unbinding (required to prevent transcription) [34].

### 3.1. Prediction of Enhanced Flexibility and siRNA Interactions of TEA-Core Dendrimers by Computer Simulations

**Figure 2:**
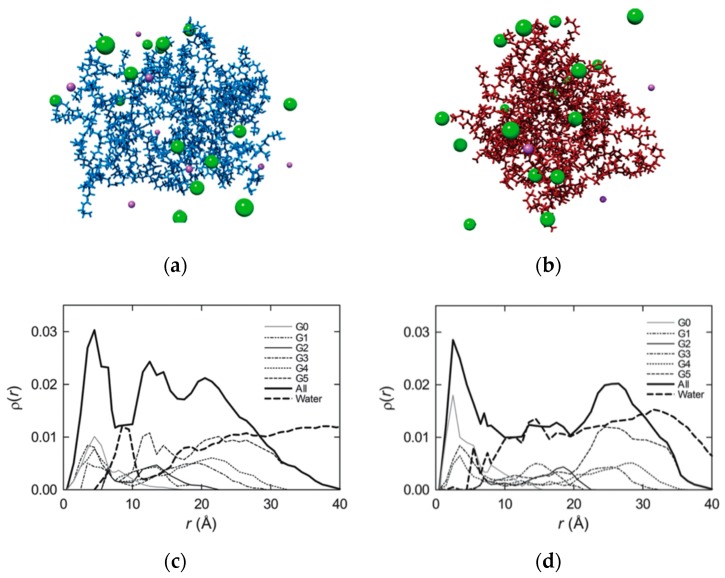
Equilibrated molecular dynamics conformations of G_5_ TEA-core (**a**) and NH_3_-core (**b**) poly(amidoamine) (PAMAM) dendrimers in physiological solution (pH = 7.4, ionic strength = 0.15 M NaCl). Each dendrimer molecule is represented as colored sticks, some ions and counterions are visualized as purple (Na^+^) and green (Cl^−^) spheres, and water molecules are not shown for clarity. Average radial monomer density *ρ*(*r*) for subsequent generations (from G_0_ to G_5_) of the TEA-core (**c**) and the NH_3_-core PAMAMs (**d**). In all cases, the origin was set at the molecular center of mass. Adapted from [37], published by RSC, 2013.

The hypothesis of diverse flexibility between TEA- and NH_3_-core based PAMAMs was verified by atomistic molecular dynamics (MD) simulations [35,36,37]. Figure 2a,b shows two MD equilibrated structures of the G_5_ TEA-core and NH_3_-core PAMAM dendrimers at a physiological pH (7.4), respectively. As intuitively perceived from these images, the TEA-core molecule is characterized by a more open conformation, with a uniform void distribution within its interior, whilst its ammonia-core counterpart is remarkably more compact, featuring a non-homogeneous, restricted void spacing. Thus, the conformation of the TEA-core PAMAM dendrimers is such that the outer branches can freely move and adjust to optimize binding with its siRNA cargo (Figure 2a). On the contrary, the more rigid and compact structure of NH_3_-core PAMAMs prevents these molecules from any induced-fit conformational readjustment, and, consequently, not all of the terminal groups are available to self-orient for optimal nucleic acid binding (Figure 2b).

Quantitative substantiation was obtained from calculating the average radial monomer density *ρ*(*r*) for each dendrimer type (shown in Figure 2c,d for subsequent dendrimer generations up to G_5_), a quantity defined as the number of atoms whose centers of mass locate within a spherical shell of radius *r* and thickness Δ*r*. Accordingly, the integration of *ρ*(*r*) over *r* yields the total number of dendrimer monomers as:

(1) $$N\left(r\right)=4\pi \int_{0}^{R}r^{2}\rho \left(r\right)dr.$$

Focusing attention on G_5_ as an example, in the case of the TEA-core molecule, the whole dendrimer *ρ*(*r*) curve (shown as a thick continuous line in Figure 2c) is characterized by the presence of two minima—the first (more pronounced) located approximately 10 Å away from the core and the second at around 17 Å—each followed by two relative maxima, at about 13 and 21 Å, respectively. These features of *ρ*(*r*) constitute a clear indication that the dendrimer core region is denser with respect to the middle–outer molecular portions (which are fairly hollow) and that the higher sub-generation monomers generate a crowded layer at the dendrimer periphery. This, in turn, accounts for the presence of a uniform distribution of hollow spaces in the central dendrimer structure, which can be filled up by a significant number of solvent molecules, as testified by the corresponding thick dashed line in Figure 2c. Considering the same data for the alternative G_5_ NH_3_-core dendrimer, the *ρ*(*r*) profile for the whole molecule (thick continuous line; Figure 2d) is representative of a complete different trend: Indeed, after the core peak, the curve quickly reaches a plateau value that spans the entire central dendrimer region and finally increases again at the molecular periphery. In other words, all dendrimer sub-generations afford a substantial contribution to the whole density curve, supporting the visual evidence (Figure 2b) of a more uniform monomer distribution within the dendrimeric structure. In line with this observation, the corresponding water density profile (thick dashed line; Figure 2d) does not feature any pronounced maximum in any specific region of the molecule but rather exhibits a uniform distribution throughout the dendrimer interior.

The postulated enhanced ability of the more flexible, extended-core (TEA) PAMAMs in interacting with siRNA molecules with respect to smaller core (NH_3_) dendrimers was next predicted by computer simulations based on the so-called molecular mechanics/Poisson-Boltzmann surface area (MM/PBSA) methodology [35,36,37,38,39,40] (see Supporting Information for detailed explanation). To this purpose, the free energy of binding normalized by the total number of charged dendrimer terminal groups (ΔG_bind_/*N*) between successive generations of the two different PAMAM-based molecules towards the siRNA sequence directed against the mRNA coding for the heat shock protein 27 (Hsp27)—a small molecular chaperone which is a vital regulator of cell survival and a major player in drug resistance—was calculated [35]. This normalization procedure was required to compare the affinity of the different dendrimer generations towards the double-stranded (ds) RNA fragment. As can be seen in Table 1, ΔG_bind_/*N* is negative for all systems considered, indicating that, under *in silico* physiological conditions (pH 7.4 and 0.15 M NaCl), the association of both dendrimeric nanovectors with their nucleic acid payloads is a thermodynamically favorable and spontaneous process. However, for each dendrimer generation, the TEA-core PAMAMs show a superior affinity for the ds-RNA sequence (i.e., ΔG_bind_/*N* more negative) with respect to their NH_3_-core counterparts. Additionally, there is a notable increase in binding strength in passing from G_4_ to G_5_, substantially ascribable to an enhanced favorable enthalpic component ΔH_bind_/*N*. This aspect accounts for the general trend of better binding and, hence, better properties as nanocarriers of high generation dendrimers, which is in agreement with experimental evidence [32] Contextually, the entropic contribution is less unfavorable (i.e., smaller) in the case of the TEA-core molecules. This lower value of –TΔS_bind_/*N* can be connected again to the enhanced flexibility and, consequently, the greater capacity of the conformational adaptation of all generations of the enlarged-core dendrimers in enwrapping the ds-RNA molecule, followed by an enhanced productive binding of the nucleic acid.

**Figure 3:**
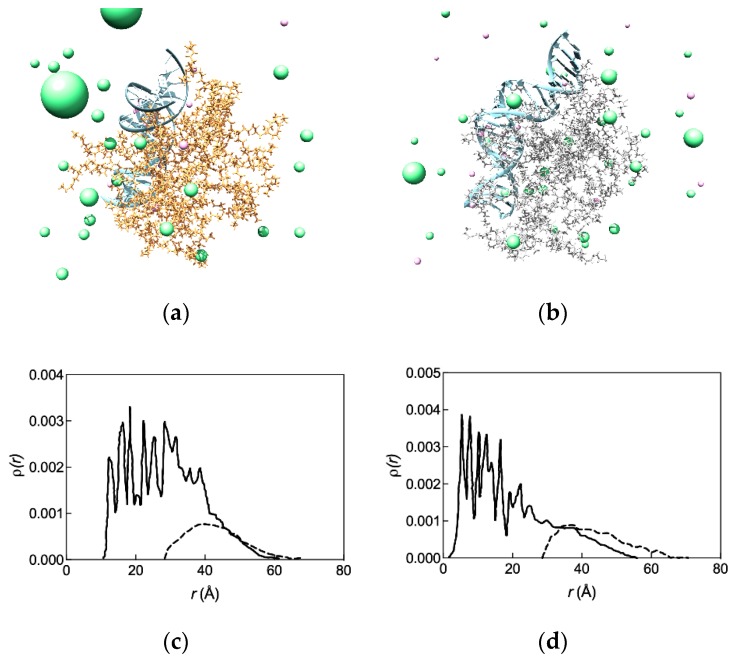
Equilibrated molecular dynamics conformations of G_5_ TEA-core (**a**) and NH_3_-core (**b**) PAMAM dendrimers in complex with Hsp27 siRNA in physiological solution (pH = 7.4, ionic strength = 0.15 M NaCl). Each dendrimer molecule is represented as colored sticks, some ions and counterions are visualized as light pink (Na^+^) and light green (Cl^−^) spheres, the siRNAs are portrayed as light blue ribbons, and water molecules are not shown for clarity. Radial density distribution *ρ*(*r*) of the dendrimer terminal nitrogen atoms in G_5_ TEA-core (**c**) and NH3-core (**d**) PAMAMs in complex with Hsp27 siRNA (continuous lines). The corresponding distributions of the siRNA phosphorous atoms in each siRNA/dendrimer complex are shown as dashed lines. Adapted from [35,36] with permission of John Wiley and Sons and Bentham Science Publishers.

The equilibrated MD snapshots of these two G_5_ dendrimer series in complex with the Hsp27 siRNA shown in Figure 3 offer additional insightful structural information. In fact, as can be inferred from Figure 3a, the conformation of the TEA-core dendrimers is such that its outer branches can readily move towards the phosphate backbone of the siRNA during complex formation so that its charged amine groups can arrange themselves via induced-fit for optimal binding with the nucleic acid. On the contrary, the more rigid and compact structure of the alternative PAMAM molecule prevents it from undergoing a significant conformational readjustment required by the induced-fit (Figure 3b); as a consequence, a smaller number of amine groups are available for optimal siRNA binding.

This differential behavior in siRNA binding is more evident when analyzing the radial density distributions of the relevant nucleic acid/dendrimer complexes reported in Figure 3c,d. For the G_5_ TEA-core dendrimer/siRNA complex, the density profiles of the primary, positively charged nitrogen atoms stretches further out towards the molecule periphery due to the electrostatic attraction of the siRNA negatively charged phosphate moieties (Figure 3c). Contextually, the density distribution of the phosphorous siRNA atoms reveals a good penetration of the nucleic acid fragment within the dendrimer outer shell. Contrarily, even in complex with siRNA the NH_3_-core G_5_, PAMAM maintains a more compact conformation that is characterized by a high degree of branch back-folding; as a result, the density of the terminal amines on the dendrimer surface is lower, and the corresponding siRNA phosphorous density distribution curve shows only a partial penetration of the nucleic acid within the dendrimer molecular structure (Figure 3d).

### 3.2. High-Generation TEA-Core PAMAM Dendrimers as Effective In Vitro and In Vivo siRNA Nanocarriers

#### 3.2.1. In Vitro Data

**Figure 4:**
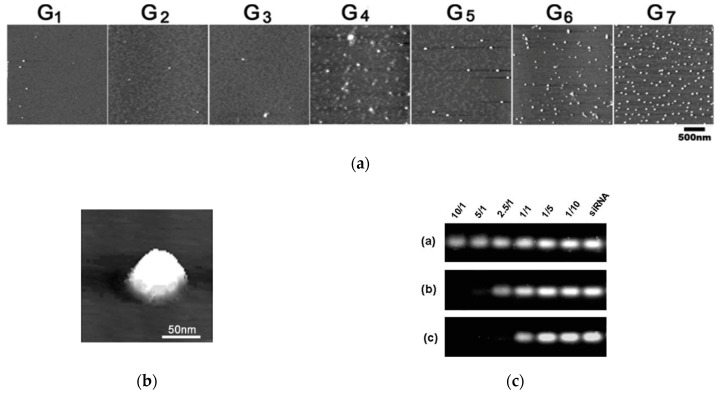
Three-dimensional atomic force microscopy (AFM) images of (**a**) Hsp27 siRNA in complex with TEA-core PAMAM dendrimers of increasing generation (**G_1_**–**G_7_**) and (**b**) a single spherical siRNA/TEA-core dendrimer (**G_7)_** complex at a final siRNA concentration of 0.0125 mg/L and at a dendrimer-to-siRNA charge ratio (N/P) of 10. (**c**) Gel retardation of Hsp27 siRNA with three different TEA-core PAMAMs ((**G_1_**) (**a**), (**G_4_**) (**b**) and (**G_7_**) (**c**)) as a function of the N/P ratio (from 10/1 to 1/10 from left to right; last lane: Naked siRNA). Adapted from [41] with the permission of the RSC.

The predicted ability of TEA-core PAMAMs to generate nanoscale siRNA/dendrimer complexes (aka dendriplexes) was experimentally verified and characterized in vitro. Figure 4a,b shows the atomic force microscopy (AFM) images of the Hsp27/dendrimer nanoparticles obtained using TEA-core dendrimers from G_1_ to G_7_ [41].

While only a few siRNA/dendrimer assemblies can be observed for smaller dendrimers (G_1_ and G_3_), an increasing number of nanoscale particles are formed with increasing dendrimer generation (Figure 4); in particular, starting from G_4_, the siRNA/dendrimer nanocomplexes progressively become more uniform, well-defined, and compact. This suggests that G_4_ is the lowest threshold TEA-core dendrimer generation for effective siRNA complexation. This assertion is substantiated by the results of the RNA mobility assay illustrated in Figure 4c: While no gel retardation effect is observed with the G_1_ dendrimer even at the maximum dendrimer-to-siRNA charge (N/P) ratio adopted (10/1), the siRNA mobility is considerably retarded by the G_4_ molecule for N/P ≥ 2.5, and, for the same N/P value, the siRNA shift in the gels is completely almost prevented by the G_7_ dendrimer [41]. The complexes formed between siRNA and high generation (≥G_4_) TEA-core PAMAMs are completely stable in physiological conditions, require strong ionic detergents such as Sodium Dodecyl Sulfate (SDS) to be disrupted and, contrarily to naked siRNA, to show considerable resistance to RNase degradation [33].

**Figure 5:**
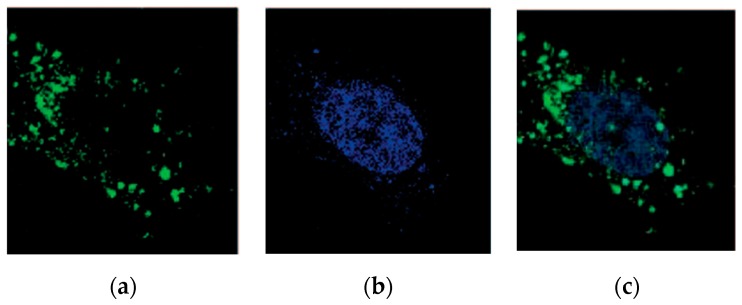
(**a**) Confocal fluorescence imaging of G_7_ TEA-core dendrimer-mediated cellular uptake of siRNA using a non-silencing siRNA sequence labeled with the green fluorescent dye Alexa 488. (**b**) Blue fluorescence images of a nucleus of the same cells stained by TOPRO-3. (**c**) Merged fluorescent images of a and b confirming the exclusive cytoplasm localization of the dendrimer/siRNA complexes. Adapted from [42] with permission of John Wiley and Sons.

The rapid and efficient cellular uptake of siRNA/TEA-core dendrimer nanoassembly was demonstrated using the human castration-resistant prostate cancer (CRPC) PC-3 cell line by confocal fluorescence imaging. In these experiments, a non-silencing (i.e., scrambled) Hsp27 siRNA sequence labeled with the green fluorescent dye Alexa 488 was employed [42]. Figure 5a shows that, 4 h after treatment, cells are populated by the green fluorescent siRNA–dendrimer nanoparticles which reside exclusively in the cell cytoplasm (Figure 5b,c). Contextually, after cell treatment with a similar amount of Alexa 488-labelled naked siRNA, no green fluorescent nanoparticles are detected inside the cells.

**Figure 6:**
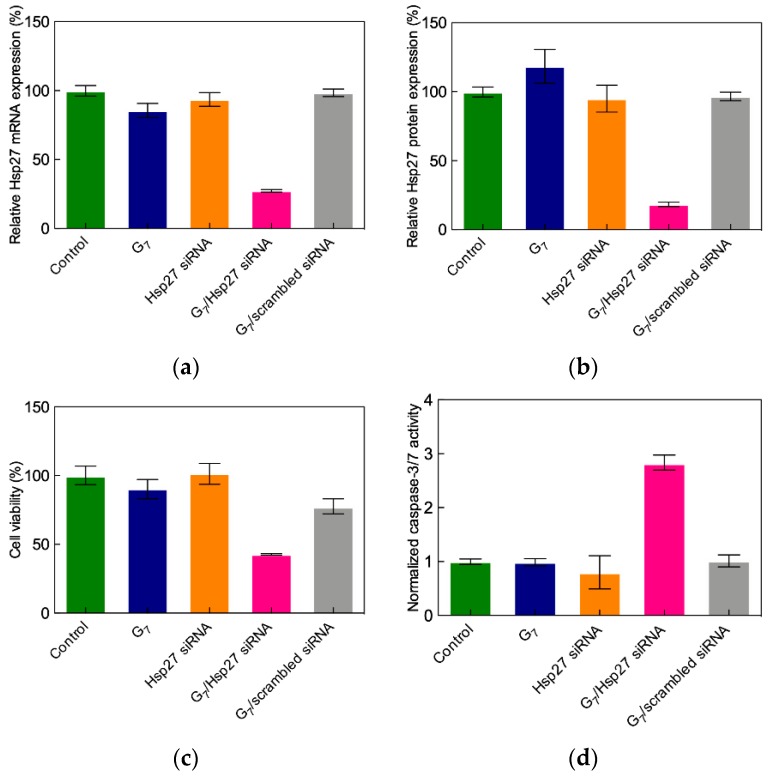
Quantitative analysis of (**a**) Hsp27 mRNA levels (determined by quantitative reverse transcription polymerase chain reaction (RT-qPCR)), (**b**) Hsp27 protein expression (determined by western blot analysis), (**c**) cell proliferation (determined by the 3-(4,5-dimethylthiazol-2-yl)-2,5-diphenyl tetrazolium bromide (MTT) assay), and (**d**) caspase-3/7 activity (measured by a colorimetric assay) in prostate cancer-3 (PC-3) cells three days after the TEA-core G_7_-mediated delivery of 50 nM Hsp27-targeting siRNA (N/P = 10) (hot pink bars in all panels). Data for dendrimer G_7_ alone (blue bars), naked Hsp27 siRNA (orange bars), and scrambled siRNA–G_7_ complexes (gray bars) are shown for comparison. Values are expressed as % relative to control (non-treated cells, green bars). Adapted from [42] with permission of John Wiley and Sons.

The successful siRNA delivery and specific gene silencing of these flexible TEA-core PAMAMs was next validated in the first proof-of-concept (POC) study targeting again Hsp27 PC-3 cells [42]. Prostate cancer is the second most commonly occurring cancer in men and the fourth most commonly occurring cancer overall; although most patients initially respond well to first-line hormone-based therapies associated with androgen ablation, after a very short time period (≈2 years), they unfortunately relapse [43], and no effective therapeutic regimen is available to treat this progressive condition. Therefore, anticancer treatments based on targeting survival genes (e.g., Hsp27) using RNAi constitute an interesting option in contrasting CRPCs [44]. The results from the POC study (Figure 6) show that transfecting PC-3 cells with TEA-core G_7_/Hsp27 siRNA complexes yields the potent, specific, and long-lasting downregulation (>50% silencing after five days) of both targeted mRNA and protein [42].

Following the knockdown of Hsp27 in PC-3 cells by the G_7_ TEA-core PAMAM dendrimer/siRNA complex, a remarkable anti-proliferative effect is observed (Figure 6c), in agreement with previous results from Rocchi et al. [45], thus showing that the inhibition of Hsp27 protein expression negatively affects PC-3 cell survival.

The main mechanism beyond this cellular effect was next demonstrated to proceed via caspase-dependent induced apoptosis. Apoptosis is programmed cell death that involves the controlled dismantling of intracellular components while avoiding inflammation and damage to surrounding cells. Apoptotic caspases are a family of endoproteases that provide critical links in cell regulatory networks controlling cell death. Specifically, the activation of these enzymes results in the inactivation/activation of substrates, and the generation of a cascade of signaling events permitting such controlled demolition of cellular components. As seen in Figure 6d, a three-fold increase in caspase-3/7 activity after Hsp27 siRNA/G_7_ TEA-core dendrimer complex treatment relative to controls is detected, ultimately confirming that Hsp27 siRNA delivered by the G_7_ TEA-core dendrimer is very effective in inhibiting Hsp27 expression and thereby inducing caspase-dependent anticancer activity in human CRPC PC-3 cells.

The dependence of the TEA-core PAMAM dendrimer-mediated siRNA silencing effect on siRNA concentration, dendrimer generation, N/P ratio and incubation time for transfection was also investigated [42]. A clear dose-dependent gene silencing was observed using the G_7_ TEA-core PAMAM nanocarrier, in line with a RNAi-mediated gene silencing mechanism. Moreover, in agreement with the molecular simulation results (see Table 1), the gene silencing efficacy was seen to increase with dendrimer generation, the G_7_ molecule being the best delivery nanovector for Hsp27 siRNA in cell-based assays. With this high generation dendrimer, optimal gene silencing was achieved at an N/P value of 10, this siRNA/dendrimer charge ratio likely providing not only the best nucleic acid degradation protection but also the most effective siRNA deployment into the cellular cytoplasm by virtue of a very efficient endosomal escape mechanism. Finally, an incubation time for transfection of 24 h was determined as the optimal condition for G_7_ TEA-core PAMAM dendrimer-mediated siRNA delivery to CRPC PC-3 cells for Hsp27 gene silencing.

**Figure 7:**
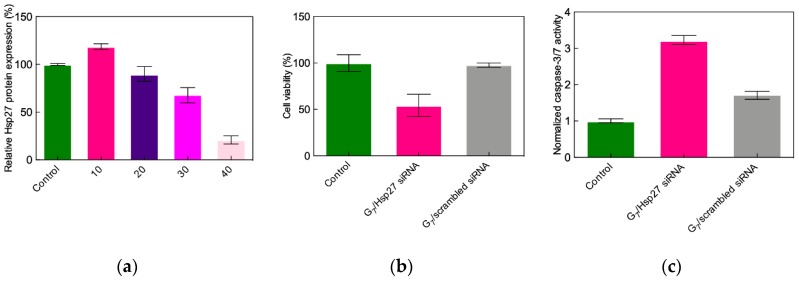
(**a**) Effect of N/P ratio on Hsp27 gene silencing, (**b**) inhibition of cell growth, and (**c**) caspase-3/7 activity in PC-3 cells three days after TEA-core G_7_-mediated delivery of 50 nM Hsp27-targeting siRNA in the presence of 10% serum (hot pink bars in all panels). Data for scrambled siRNA–G_7_ complexes (gray bars) are shown for comparison. Values are expressed as % relative to control (non-treated cells, green bars). Adapted from [42], with permission of John Wiley and Sons.

Sensitivity to serum proteins constitutes an Achilles’ heel of cationic nanovectors in siRNA delivery. In fact, since high N/P ratios are always required for efficient nucleic acid transfection (as discussed above), the resulting overall charge of the delivery complexes is positive. As such, electrostatic forces may drive their interaction with the negatively charged serum proteins, this process eventually resulting in the disintegration of the nanoassemblies with consequent premature nucleic acid release and degradation. The lead G_7_ TEA-core PAMAM dendrimer was indeed not exempt from this drawback, as no Hsp27 gene silencing was observed in the presence of 10% serum with G_7_ nanovectors loaded with 50 nM Hsp27 siRNA at the optimal, serum-free N/P ratio of 10. In trying to overcome these negative results, gene silencing experiments were performed at progressively increasing dendrimer-to-siRNA ratios. Potent Hsp27 gene silencing is indeed reached in PC-3 cells at N/P = 40, supported by a strong inhibition of cell proliferation and high caspase-3/7 activation, as shown in Figure 7. Pleasingly, the potent in vitro anticancer activity of the G_7_ TEA-core PAMAM-based nanovectors was paralleled by the complete absence of toxicity, as assayed by 3-(4,5-dimethylthiazol-2-yl)-2,5-diphenyl tetrazolium bromide (MTT) tests for cell viability and by lactate dehydrogenase (LDH) release for cell membrane damage [42]. Finally, no acute toxicity was observed in preliminary tests conducted by treating healthy mice via a tail vein injection with scrambled siRNA–G_7_ complexes, naked Hsp27 siRNA, and the G_7_ TEA-core dendrimer alone, as well as a glucose solution as control.

In aggregate, these findings supported the concept that the flexible, high-generation TEA-core PAMAM dendrimers could be effective nanovectors for in vitro siRNA delivery.

#### 3.2.2. In Vivo Data

Encouraged by the results obtained in vitro, high-generation TEA-core dendrimers were tested for in vivo gene silencing performance in various cancer models, including prostate [46], ovarian [47], liver [48], and glioblastoma [49,50]. The application to ovarian cancer treatment represents a particularly interesting example, since, beside the remarkable silencing of AKT—a gene activated in 36% of primary tumors, with the activation being associated with high-grade cancer and aggressive clinical behavior, protection to induced apoptosis, drug resistance and disease relapse [51]—the combined administration of the anticancer drug paclitaxel with AKT siRNA delivered using G_6_ TEA-core PAMAM nanovectors resulted in a synergistic therapeutic effect in vivo.

**Figure 8:**
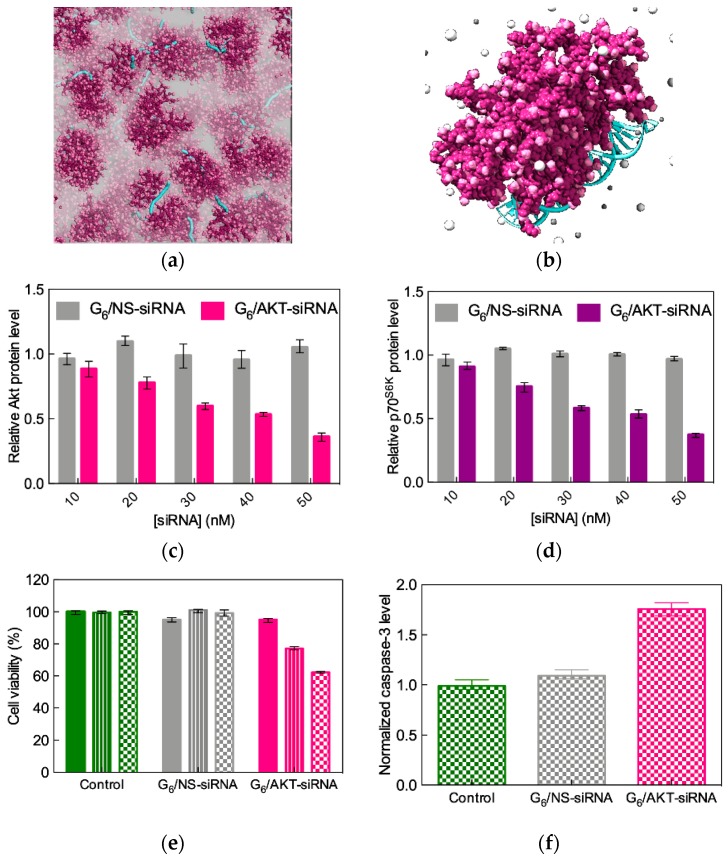
(**a**) Simulation of G_6_ TEA-core PAMAM dendrimers in complex with AKT siRNA at N/P = 5. The dendrimers are portrayed as wine-colored spheres, with charged amine groups depicted in pink. siRNA molecules are shown as turquoise sticks. A transparent gray field is used to represent the solvent environment. (**b**) Zoomed view of one single G_6_ TEA-core PAMAM molecule in complex with one AKT siRNA (colors as in panel a). Some Cl^-^ and Na^+^ counterions are shown as white and light gray spheres, respectively; water molecules are not shown for clarity. siRNA concentration-dependent inhibition of AKT (**c)** and its downstream effector p70^6SK^ (**d**) in SKOV-3 cells three days after TEA-core G_6_-mediated delivery (N/P = 5). Data for non-specific (NS) siRNA–G_6_ complexes are shown for comparison. Protein expression levels were determined by western blotting, quantified by densitometry, and are expressed as fold-change normalized to β-actin. (**e**) Time-dependent growth inhibition of SKOV-3 cells transfected with G_6_ TEA-core PAMAM dendrimers (N/P = 5) and with non-specific (NS) siRNA–G_6_ complexes, as determined by the MTT assay. Values are expressed as % relative to control (non-treated cells). Filled bars: 24 h post transfection (p.t.), striped bars: 48 p.t., checked bars: 72 h p.t. (**f)** Caspase-3 activation in SKOV-3 cells determined 72 p.t. with G_6_ TEA-core PAMAM dendrimers at N/P = 5. Data for non-specific (NS) siRNA–G_6_ complexes are shown for comparison. Values are expressed as fold change normalized to β-actin used as control. Adapted from [47], which is an open access article published under an ACS AuthorChoice License.

In this study, the effective AKT gene-silencing mediated by the G_6_ dendrimer nanocarriers (Figure 8a,b) was first verified in SKOV-3 cells, a gold standard model for drug-resistant ovarian cancer, as shown in Figure 8c–f. A non-specific (NS) siRNA sequence was also used in all experiments for comparison.

As seen from Figure 8c, the G_6_ TEA-core PAMAM-mediated gene silencing effect is dependent on siRNA concentration, with approximately 70% inhibition achieved using 50 nM siRNA. Interestingly, AKT knockdown negatively affects the expression of p70^S6K^, an AKT effector protein involved in cell survival within the phosphatidylinositol 3-kinase (PI3K)/AKT pathway-driven ovarian cancer development [51]. With the same nanovector/siRNA system, the inhibition of cancer cell growth is evident after 72 h post transfection (Figure 8e), and the loss of cell viability can be ascribed to induced apoptosis, as monitored by the significant increase of caspase-3 activity (Figure 8f) [47].

**Figure 9:**
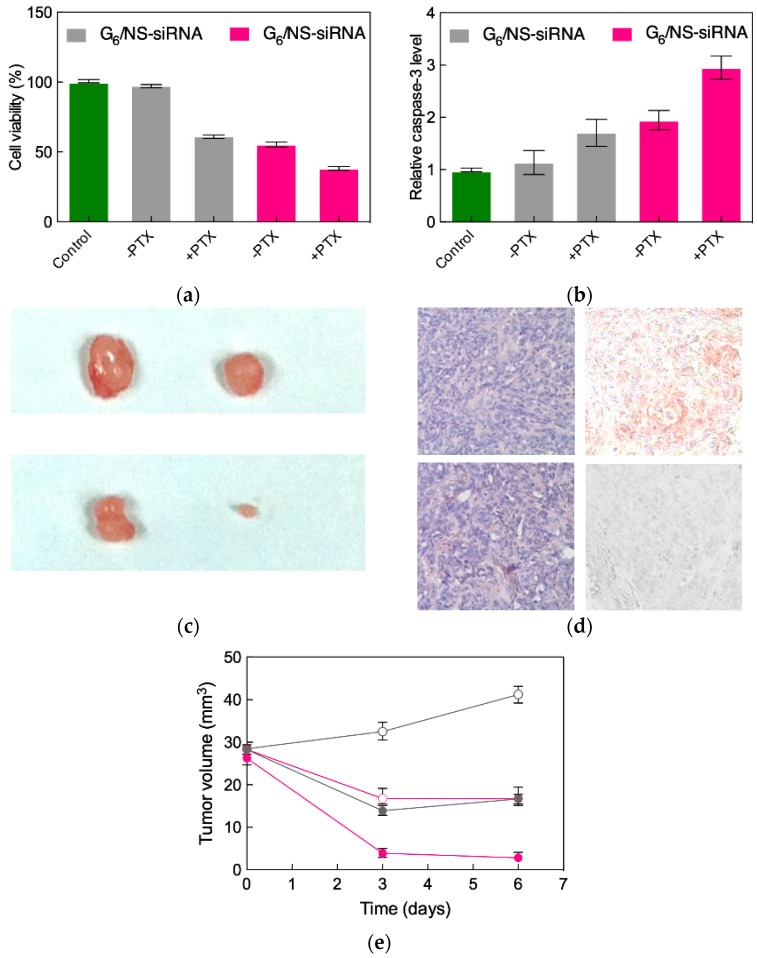
SKOV-3 cell viability (**a**) and relative caspase-3 activation (**b**) after transfection with non-specific (NS) siRNA or AKT siRNA delivered by the G_6_ TEA-core dendrimer nanovectors (N/P = 5) alone or in combination with paclitaxel (100 nM). Non-treated cells and β-actin were used as respective controls. (**c**) Tumor volumes from SKOV-3 xenografted mice treated with non-specific (NS) siRNA (top panel, left) or AKT siRNA delivered by the G_6_ TEA-core dendrimer nanovectors (N/P = 5) alone (top panel, right) or in combination with paclitaxel (100 nM, bottom panel). (**d**) Drastic reduction of AKT levels in tumor xenografts injected with AKT siRNA G_6_ TEA-core dendriplexes (**bottom, left**), and the corresponding histological sample showing sign of necrosis (**bottom, right**), compared with xenografts treated with NS siRNA delivered with the same nanovectors showing no reduction of AKT levels (**top, left**) and no necrosis (top, right). (**e**) Tumor volume during combined treatment of AKT siRNA G_6_ TEA-core dendriplexes/paclitaxel (filled hot pink circles) of SKOV-3 mice xenografts, compared with nanodelivered AKT siRNA (open hot pink circles) or paclitaxel alone (filled gray circles). Data for nanodelivered NS siRNA are shown for control (open gray circles). Adapted from [47], which is an open access article published under an ACS AuthorChoice License.

Current chemotherapeutic regimens unfortunately have limited efficacy in patients with advanced ovarian cancer [52]. Paclitaxel is the current first-line drug for treating this malignancy in the clinics, and induced apoptosis has been established as one of the main mechanisms of action of this molecule. Therefore, we speculated that, when administered together, AKT-targeting siRNA and paclitaxel might act synergistically in promoting specific cancer cell death. This hypothesis was initially verified in in vitro experiments, showing how the combined treatment of AKT siRNA/G6 TEA-core dendrimer nanoparticles substantially enhanced SKOV-3 cell growth inhibition via induced apoptosis, as illustrated in Figure 9a,b.

These promising results prompted us to proceed with in vivo experiments. Accordingly, SKOV-3 cells were subcutaneously injected in nude mice, and tumors were allowed to grow until they reached a volume of approximately 30 mm^3^. The xenografts were then first treated with intratumoral injections of either AKT siRNA/ or non-specific siRNA/G_6_ TEA-core dendriplexes in the absence of paclitaxel. The differential reduction of tumor volume is well evident in the upper panel of Figure 9c: A tumor shrinkage of ≈50% in mice administered with the targeted siRNA with respect to those receiving the non-specific treatment can be readily appreciated. Concomitantly, immunohistochemistry confirmed the drastic decrease of AKT expression in the AKT knockdown mice (Figure 9d, right panel), the corresponding tumors showing signs of necrosis (Figure 9d, left panel).

Finally, the same assays were performed in tandem with paclitaxel administration via mice intraperitoneal injection. The corresponding xenografts reveal a remarkable cooperative action of the two treatments with respect to the chemotherapeutic drug per se (Figure 9c, lower panel), with a reduction of tumor growth of 85% compared to the treatment with paclitaxel alone (Figure 9f) while the G_6_ TEA-core dendrimer or the AKT siRNA alone have no effect (data not shown). To the best of our knowledge, this constitutes the first study documenting a potentiated anticancer effect of paclitaxel while co-administered with AKT siRNA mediated by dendrimer nanovectors to ovarian cancer both in vitro and in vivo.

### 3.3. Low-Generation TEA-core PAMAM Dendrimers as Effective In Vitro and In Vivo siRNA Nanocarriers

Though the high-Generation G_6_ and G_7_ TEA-core dendrimers were very effective in delivering siRNA molecules to various cancer models in vitro and in vivo, the large-scale chemical synthesis of these molecules required for their clinical applications is laborious and very time/resource consuming (e.g., extensive dendrimer purification is intrinsically difficult, being hampered by the presence of highly similar side-products) [53]. These issues imply that the good manufacturing practice (GMP) claimed for products expected to undergo clinical trials is technically very challenging. Therefore, finding a way to endow lower generation dendrimers with effective and efficient siRNA delivery could be a worthy goal per se, as discussed below.

#### 3.3.1. Functional Delivery of Sticky siRNA

In 2007, Jean Paul Behr and his group showed that small interfering RNAs bearing short complementary A_n_/T_n_ (*n* = 5–8) sticky overhangs delivered using polyethyleneimine (PEI) result in enhanced gene silencing with respect to standard siRNAs [54]. Since PEI is one of the best non-viral DNA carriers but its efficiency drops dramatically during siRNA transfection [55], its ability to quantitatively deliver sticky siRNAs could be attributed only to the presence of the complementary A_n_/T_n_ overhangs. According to Behr’s explanation, the latter can self-assemble into gene-like, long double-stranded RNA, and this in turn allows for the successful cellular delivery by PEI by virtue of a mechanism utterly similar to that governing plasmid DNA transfection. On the other hand, we reasoned that an additional contributing factor to the enhanced gene silencing of nano-delivered ssiRNAs could also be related to the inherent flexibility of the terminal, single-strand nucleic acid fragments; this might allow them to behave as clamps that, just like protruding molecular arms, can better enwrap and tightly hold the nanovector, thereby enhancing binding and, ultimately, delivery. Accordingly, we set on to verify these concepts with the ultimate purpose of exploiting low generation TEA-core PAMAM dendrimers as efficient nanocarriers in RNAi.

#### 3.3.2. In Vitro Preliminary Data of Sticky siRNA Delivery by Lower Generation TEA-Core PAMAMs

Under this perspective, we initially constructed two sticky siRNA molecules with complementary A_5_/T_5_ and A_7_/T_7_ 3′-overhangs to target Hsp27 and TCTP (a highly conserved protein present in all eukaryotic organisms that regulate cell survival in human tumors) in prostate and breast cancer models [56,57]. In parallel, we also synthesized four additional non-complementary ssiRNAs (i.e., A_5_/A_5_, A_7_/A_7_, T_5_/T_5_, and T_7_/T_7_) to investigate the effect of chemistry, length and non-complementarity on the relevant gene silencing potential with respect to the standard (A_2_/T_2_) siRNAs discussed above [56,57].

**Figure 10:**
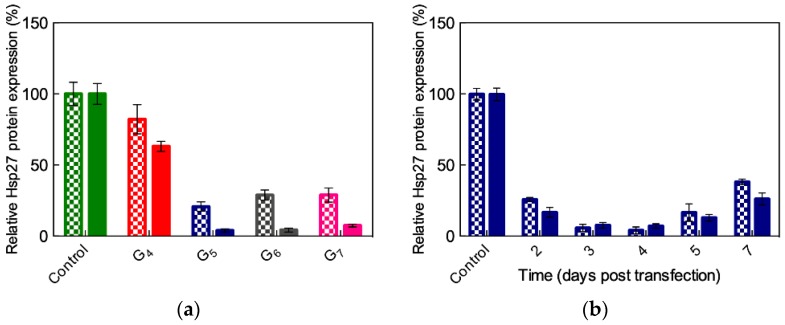
(**a**) Hsp27 gene silencing upon delivery of complementary ssiRNAs mediated by different generations of TEA-core PMAMAM to PC-3 cells. Checked bars: Data for A_5_/T_5_ ssiRNA; solid bars: Data for A_7_/T_7_ ssiRNA. In these experiments, vinculin was used as reference and non-treated cells were used for control. (**b**) Long-term Hsp27 silencing achieved with A_5_/T_5_ and A_7_/T_7_ ssiRNAs (50 nM) delivered by G_5_ TEA-core PAMAMs at N/P = 10. Redrawn from [56], with permission of the American Chemical Society.

Next, we preliminarily tested our TEA-core dendrimers from Generation 4 to Generation 7 for their ability to deliver complementary ssiRNAs in PC-3 cells. Figure 10a shows that, contrarily to conventional A_2_/T_2_ siRNA delivery, for which Generation 6 or (better) 7 dendrimers were requested to achieve biological effects (see § 3.2, [42,47]), a significant gene silencing can be achieved in PC-3 cells starting from the G_5_ nanocarriers, the A_7_/T_7_ ssiRNA being somewhat more effective than the A_5_/T_5_ counterpart for all nanovector generations (Figure 10a). Since G_5_ was the lowest and most effective dendrimer generation for the in vitro delivery of ssiRNAs, further investigations were carried out using this nanovector. In particular, the best, long-term gene silencing was obtained with the G_5_ TEA-core assisted delivery of 50 nM ssiRNas at N/P ratio = 10, as highlighted in Figure 10b.

#### 3.3.3. *In Silico* Binding Affinity of ssiRNAs with G_5_ TEA-Core Dendrimer Nanovectors

**Figure 11:**
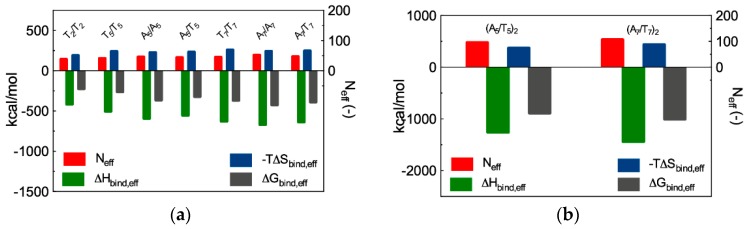
Total effective free energy (ΔG_bind,eff_ = ΔH_bind,eff_–TΔS_bind,eff_), enthalpic (ΔH_bind,eff_), and entropic (–TΔS_bind,eff_) components for the binding of (**a**) ssiRNAs featuring complementary and non-complementary overhangs of different length and (**b**) dimeric ssiRNAs with the G_5_ TEA-core PAMAM dendrimer. N_eff_ is the number of effective dendrimer positive charges involved in nucleic acid binding (see Table A1 and Table A2 in Appendix A and text for more details). (**b**) Redrawn from [57], with permission of the American Chemical Society.

**Figure 12:**
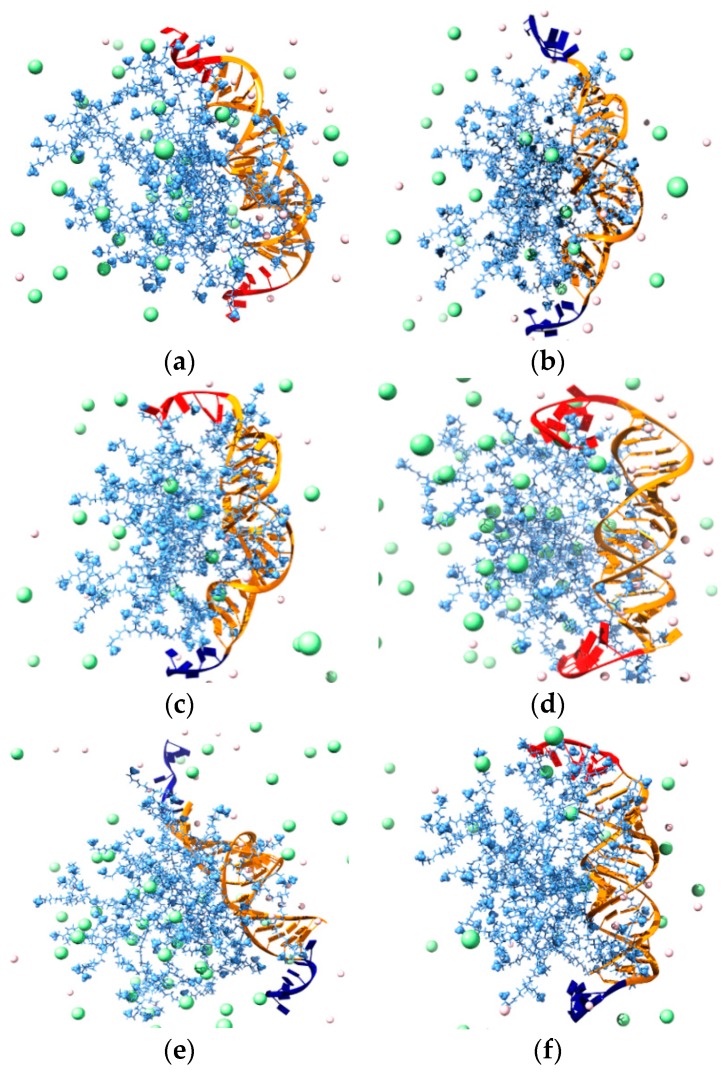
Examples of equilibrated molecular dynamics (MD) snapshots of G_5_ TEA-core dendrimer in complex with A_5_/A_5_ (**a**), T_5_/T_5_ (**b**), A_5_/T_5_ (**c**), A_7_/A_7_ (**d**), T_7_/T_7_ (**e**), and A_7_/T_7_ (**f**) ssiRNAs at pH 7.4 and in the presence of 0.15 M NaCl. In all panels, the dendrimer is shown as cornflower blue sticks, and the terminal charged amine groups are highlighted as sticks-and-balls. The ssiRNA is portrayed as an orange ribbon, with the two overhangs (A_n_) and (T_n_) colored in red and navy blue, respectively. Some Cl^−^ and Na^+^ ions and counterions are shown as light green and light pink spheres, respectively. Water molecules are not shown for clarity. Redrawn from [57], with permission of the American Chemical Society.

Before embarking in further time- and resource-consuming experimental investigations, we performed MD simulations to predict and understand if and how the different ssiRNA overhangs could impact G_5_ TEA-core mediated delivery. The *in silico* investigation started by verifying our own hypothesis, according to which the protruding, flexible overhangs could promote a better interaction and stronger binding of monomeric ssiRNAs to their dendrimeric nanocarriers via molecular dynamics simulations. The MD results are summarized in Figure 11a (see Table A1 in Appendix A for full MD results), while some exemplificative images extracted from the corresponding equilibrated MD trajectories are shown in Figure 12.

The computer simulations reveal that both the nature of the overhangs and their length influence the interaction of the relevant ssiRNAs with the G_5_ dendrimer nanocarrier. For the first aspect, the first three columns in Table A1 show that ssiRNAs with A_n_/A_n_ overhangs are characterized by the most favorable free energy of binding values (ΔG_bind_ = −409.9 kcal/mol for A_5_/A_5_ and −447.9 kcal/mol for A_7_/A_7_), followed by the ssiRNAs bearing complementary overhangs (ΔG_bind_ = -387.4 kcal/mol for A_5_/T_5_ and -422.9 kcal/mol for A_7_/T_7_), and, last, by the ssiRNAs with T_n_/T_n_ overhangs, which are characterized by the lowest nanovector affinity (ΔG_bind_ = −316.8 kcal/mol for T_2_/T_2_, −344.8 kcal/mol for T_5_/T_5_, and −402.2 kcal/mol for T_7_/T_7_, respectively). Concerning the second aspect, these data clearly indicate that the presence of longer overhangs enhances nanovector/nucleic acid binding, the ssiRNA with the shorted overhangs T_2_/T_2_ being the one with the lowest ΔG_bind_ value in the entire series.

Further interesting data were obtained by calculating the effective free energy of binding (ΔG_bind,eff_), i.e., the specific energetic contribution to ssiRNA/nanocarrier complex formation afforded only by those dendrimer branches in constant and productive interaction with its nucleic acid cargo. An analysis of each ssiRNA/dendrimer nanoassembly MD simulation allowed us to precisely identify and quantify these dendrimer residues (N_eff_); next, a per residue decomposition of the total binding free energy (see Supporting Information for details) led to the corresponding values of ΔG_bind,eff_ (Figure 11a and Table A1). The first notable result regards N_eff_: Indeed, not only the smallest number of dendrimer branches involved in nanovector binding (38) pertains to the nucleic acid fragment with the shortest overhangs (T_2_/T_2_), but also the values of N_eff_ follow a clear increasing trend from T_n_/T_n_ to A_n_/A_n_ to A_n_/T_n_ ssiRNAs. Concomitantly, the corresponding ΔG_bind,eff_ values become more favorable (i.e., more negative) in the same order.

The deconvolution of ΔG_bind,eff_ in its enthalpic (ΔH_bind,eff_) and entropic (−TΔS_bind,eff_) components (Figure 11a and Table A1) reveals that the nanovector/siRNA interaction is prevalently enthalpic in nature, although entropic effects linked to the released of ions, counterions, and water molecules in the bulk solvent upon complex formation also contribute in modulating the individual intermolecular affinities. Thus, taking the ssiRNAs series bearing five-nucleotide long overhangs as an example, it is easily seen that the A_5_/A_5_ ssiRNA has both the most favorable enthalpic contribution (ΔH_bind,eff_ = −592.0 kcal/mol) and the least unfavorable entropic term (−TΔS_bind,eff_ = 227.2 kcal/mol) in the homologous series. The best results for the A_5_/A_5_ ssiRNA/G_5_ TEA core PAMAM complex can be rationalized, from the enthalpic viewpoint, by taking into account the high number of favorable electrostatic interactions, supported by the greatest value of N_eff_ for this homologous series (46), along with other non-bonded, stabilizing contacts between the nanovector and the nucleic acid, including its overhangs (see Figure 12a). From the entropic perspective, the more rigid nature and the enhanced clamping propensity of the A-based overhangs with respect to the T-based ones translate into more permanent and effective contacts between the full nucleic acid fragments (overhangs included) and the positively charged dendrimer terminal groups (Figure 12a). The least performing nanoassembly in this series, i.e., the one involving the T_5_/T_5_ ssiRNA, is characterized by the smaller value of N_eff_ (41), the lowest enthalpic variation (ΔH_bind,eff_ = −503.1 kcal/mol) and the most unfavorable entropic component (−TΔS_bind,eff_ = 241.1 kcal/mol). This latter term is quickly understood, considering the remarkable flexibility of the T_n_ protruding overhangs, which fluctuate in the solvent for most of the time of the corresponding MD trajectory (Figure 12b). When interacting with the nanovector terminal groups, these overhang molecular movements are frozen, resulting in a considerable loss of degrees of freedom and, hence, entropic penalty. As a further effect, the corresponding ssiRNA/nanocarrier opposite-charge contacts are suboptimal, and this directly reflects in a decrease of ΔH_bind,eff_. The ssiRNA with complementary overhangs A_5_/T_5_ exhibits an intermediate behavior (−ΔH_bind,eff_ = −554.9 kcal/mol and −TΔS_bind,eff_ = 233.4 kcal/mol), stemming from a compensatory effect between the rigid A_n_ arm (with high binding tendency) and the springy T_n_ arm (endowed with less efficient dendrimer clasping propensity), as illustrated in Figure 12c. An utterly analogous situation—governed by the same molecular factors described above—is observed for the homologous ssiRNA series bearing longer overhangs, for which the affinity towards the G_5_ TEA-core dendrimer increases in the order A_7_/A_7_ > A_7_/T_7_ > T_7_/T_7_ (Figure 11 and Figure 12d–f, Table A1). These results allowed us to draw some general considerations about the effect of nature and lengths of the ssiRNA overhangs on their interaction with the G_5_ TEA-core dendrimer, as follows. First, longer overhangs are more beneficial to nanovector/ssiRNA interactions than shorter ones by virtue of the higher number of dendrimer residues (N_eff_) in permanent and efficient contact with the nucleic acid fragment. In addition, for a given length of non-complementary overhangs, the more flexible nature of the T_n_ sequence is detrimental to nanoassembly formation with respect to the alternative A_n_ strand, since the relevant less-optimized nanoparticle structure and the larger entropic penalty paid upon dendrimer/ssiRNA complex formation result in a lower affinity of the nucleic acid fragment for its nanovector.

**Figure 13:**
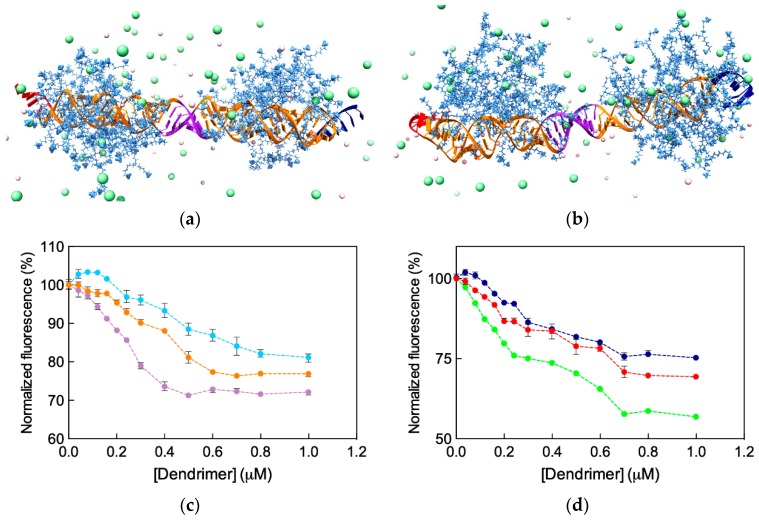
Equilibrated MD snapshots of the (A_5_/T_5_)_2_ (**a**) and (A_7_/T_7_)_2_ (**b**) dimeric ssiRNAs in complex with the G_5_ TEA-core dendrimer pH 7.4 and in the presence of 0.15 M NaCl. Molecule representations and color scheme as in Figure 12. The double-stranded portion of the concatenated (hybridized) ssiRNAs is highlighted in purple. (**c**) and (**d**) Experimental binding of ssiRNAs bearing complementary and non-complementary overhangs with the G5 TEA-core dendrimer by ethidium bromide (EB) displacement assay. Color legend: (**c**) Light blue, T_5_/T_5_ ssiRNA; orange, A_5_/A_5_ ssiRNA; light purple, A_5_/T_5_ ssiRNA; dark blue; (**d**) T_7_/T_7_ ssiRNA; red, A_7_/A_7_ ssiRNA; light green, A_7_/T_7_ ssiRNA. Adapted from [57], with permission of the American Chemical Society.

The next part of the *in silico* investigation was devoted to verify the second hypothesis, according to which ssiRNAs could self-assemble into gene-like structures via the formation of hybrid bridges between the complementary overhang sequences and, in doing so, enhance their affinity for nanovectors. In his original work [54], Behr already showed that this oligomerization or concatenation process enhanced cooperative and multivalent PEI/A_8_/T_8_ ssiRNA interactions, thereby leading to better delivery efficiency. Most importantly, however, since no concatemers were detected in the absence of nanovectors (i.e., PEI or G_5_ TEA-core PAMAM dendrimers), we further reasoned that the nanocarriers themselves must play an active role in directing encounters between individual ssiRNA/nanovector complexes, thus promoting complementary overhang concatemerization. To assess these concepts, we performed further MD simulations on G_5_ TEA-core PAMAMs in complex with two dimeric ssiRNAs, (A_5_/T_5_)_2_ and (A_7_/T_7_)_2_ (see Figure 13a,b). The computational results are graphically reported in Figure 11b (and numerically listed in Table A2). When comparing these data with those relative to the monomeric ssiRNAs (i.e., A_5_/T_5_ and A_7_/T_7_, Figure 11a and Table A1), some important information can be immediately appreciated. First, the number of nanovector-charged branches in productive contact with the nucleic acid are larger than twice the sum of the value predicted for the analogous monomeric nanoassemblies (i.e., N_eff_ = 96 for (A_5_/T_5_)_2_ and (2 × 44) = 88 for A_5_/T_5_, and N_eff_ = 107 for (A_7_/T_7_)_2_ and (2 × 47) = 94 for A_7_/T_7_, respectively, Table A3). This enhancement of stabilizing intermolecular contacts for the concatenated systems can be ascribed to the presence of the extra double-stranded portion of the hybridized ssiRNAs (Figure 13a,b), which, being more rigid and globally more negatively charged than the single stranded overhangs, induces a further conformational adaptation of the dendrimer terminal units to accommodate a larger number of favorable electrostatic interactions.

The synergistic effect of ssiRNA concatemerization is also evident in the corresponding binding thermodynamics. Indeed, both ΔG_bind,eff_ and ΔH_bind,eff_ for the hybridized ssiRNAs are more favorable than two times the corresponding values for the monomeric ssiRNAs, while the decrease in the entropic contributions (–TΔS_bind,eff_) for the dimeric ssiRNAs is less disfavoring for the former systems with respect to twice the values for the latter ones, as summarized in Table A3.

All these data indeed provide a computational support to the idea that dimeric ssiRNAs generated by nucleic acid fragments bearing complementary overhangs which might hybridize into a central (A_n_/T_n_)_2_ double-stranded portion result in a synergistic binding with the G_5_ TEA-core dendrimer nanovector with respect to siRNAs characterized by both short and/or non-complementary overhangs. Pleasingly, these theoretical predictions were confirmed by ethidium bromide (EB) displacement fluorescence spectroscopy assays (Figure 13c,d), according to which the experimental binding affinity of the different ssiRNAs for the dendrimer follows exactly the same order anticipated by simulations, that is: A_n_/T_n_ > A_n_/A_n_ > T_n_/T_n_.

**Figure 14:**
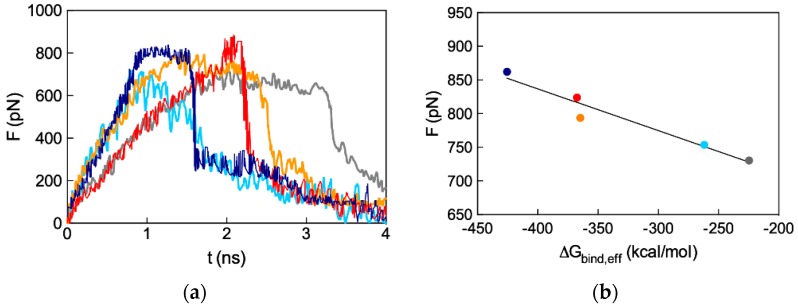
(**a**) Profiles of the average force required to unbind ssiRNAs from their G_5_ TEA-core dendrimer nanovectors as obtained from steered molecular dynamics (SMD) simulations. Color legend: Dark blue, (T_7_/T_7_) ssiRNA; light blue, (T_5_/T_5_) ssiRNA; gray, (T_2_/T_2_) (i.e., non-sticky) siRNA; red, (A_7_/A_7_) ssiRNA; orange, (A_5_/A_5_) ssiRNA. (**b**) Relationship between the SMD peak force and the corresponding effective free energy of binding ΔG_bind,eff_ for the corresponding ssiRNA and the G_5_ dendrimers. Redrawn from [57], with permission of the American Chemical Society.

The final step of the *in silico* study concerned another fundamental aspect in nanovector-assisted effective siRNA delivery and gene silencing—the disassembly of the nanocomplexes in the cellular cytoplasm to make the nucleic acid cargo available to the RNAi machinery. To the purpose, advanced computational techniques based on steered molecular dynamics (SMD) simulations were applied to the four complexes formed by ssiRNAs bearing non-complementary overhangs, as well as by the standard (T_2_/T_2_) siRNA and the G_5_ TEA-core dendrimer (see Supplementary Materials)). Briefly, during SMD runs, each siRNA molecule was drifted away from its nanocarrier using a constant pulling speed, and the behavior of the force require to break the corresponding complexes was recorded as a function of time. The results from SMD simulations are shown in Figure 14a, from which it is seen that the peak force that needs to be exerted to dissociate the nucleic acids fragments from their nanocarrier increases in the order: 730 pN for T_2_/T_2_, 753 pN for T_5_/T_5_, 794 pN for A_5_/A_5_, 824 pN for T_7_/T_7_, and 862 pN for A_7_/A_7_. If the nanovector/ssiRNA disassembly force is plotted against the corresponding effective formation free energy (ΔG_binf,eff_) value (Figure 11a and Table A1), a linear relationship is obtained (Figure 14b, R^2^ = 0.95), indicating that the tighter the ssiRNA/dendrimer binding, the stronger the force required to disassemble the corresponding complex. In other words, a very high affinity between nanocarrier and cargo, although useful for protection and transport, will ultimately be detrimental to the final step, i.e., efficient release; accordingly, the ideal system must represent the best compromise among these counteracting effects.

#### 3.3.4. In Vitro Delivery of ssiRNAs with G_5_ TEA-Core Dendrimer Nanovectors

**Figure 15:**
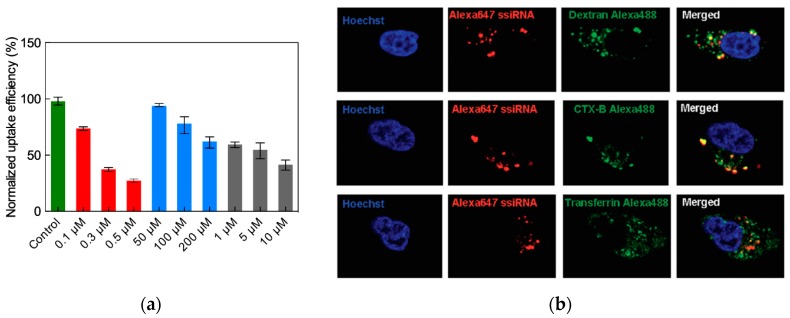
(**a**) Effect of cytochalasin D (a macropinocytosis inhibitor, red bars), genistein (a caveolae-mediated endocytosis inhibitor, light blue bars), and chlorpromazine (a clathrin-mediated endocytosis inhibitor, gray bars) on the uptake of Alexa 647-labelled A_5_/T_5_ ssiRNA/G_5_ TEA-core dendrimer nanoparticles by PC-3 cells. Values are normalized to Alexa 647-labeled ssiRNA/G_5_ TEA-core dendrimer nanoparticles uptake in the absence of any inhibitor. (**b**) Colocalization of the Alexa 647-labelled A_5_/T_5_ ssiRNA/G_5_ TEA-core dendrimer nanoparticles with different endocytosis biomarkers: Top panel, dextran (macropinocytosis biomarker); middle panel, cholera toxin B (caveolae-mediated endocytosis biomarker); bottom panel, transferrin (clathrin-mediated endocytosis biomarker).

The uptake of ssiRNA/G_5_ TEA-core PAMAM dendriplexes by PC-3 cells was first verified using live-cell confocal microscopy that confirmed both efficient internalization and cytoplasmic localization of the nucleic acid-loaded nanocarriers. Since the mechanism presiding cellular uptake of nanoparticles can involve several pathways, including macropinocytosis, clathrin-mediated endocytosis, and caveolae-mediated endocytosis [58], the mechanism of uptake was investigated using specific inhibitors and biomarkers of various endocytic pathways. As an example, Figure 15a shows that a meaningful reduction of G_5_ TEA core dendrimer/A_5_/T_5_ ssiRNA complexes was achieved only in the presence of the macropinocytosis inhibitor cytochalasin D, while only very weak effect was obtained in the presence of the two alternative inhibitors, that is genistein (an inhibitor of caveolae-mediated endocytosis) and chlorpromazine, a clathrin-mediated uptake specific blocker. In addition, a robust colocalization of the Alexa 647-labelled nanoparticles with dextran (a prototypic macropinocytosis biomarker) was observed, while minor-to-moderate colocalization was evidenced using either the transferrin of cholera toxin B (biomarkers for clathrin- and caveolae-mediated endocytosis) (Figure 15b).

**Figure 16:**
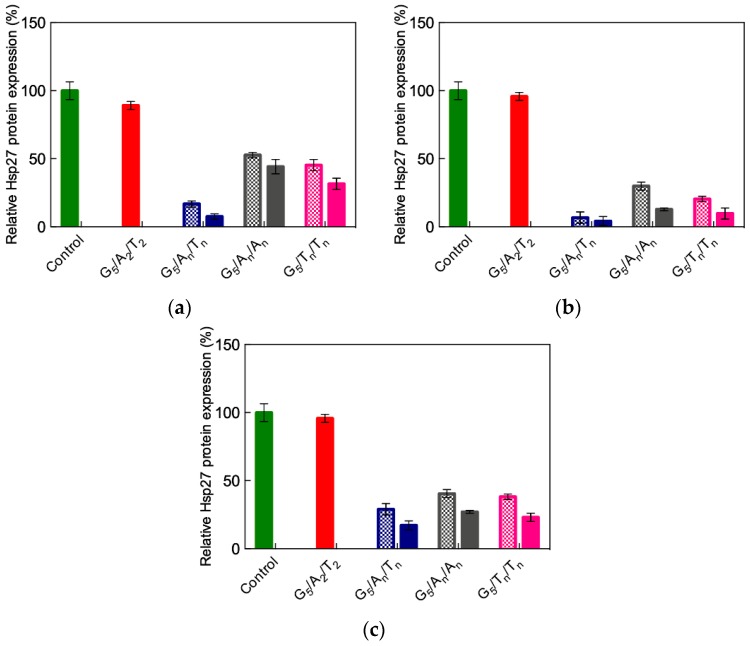
Hsp27 gene silencing upon delivery of different ssiRNAs (50 nM) mediated by G_5_ TEA-core PAMAM (N/P = 10) to PC-3 cells (**a**), MDA-MB-231 cells (**b**), and MCF-7 cells (**c**). Checked bars: Data for ssiRNA with n = 5; solid bars: Data for ssiRNA with n = 7. In these experiments, vinculin was used as reference, and non-treated cells were used for control (green solid bar). Data for non-sticky siRNA (A_2_/T_2_) are also shown for comparison (red solid bar). MDA-MB-231 and MCF-7 are two different breast cancer cell lines. Redrawn from [57], with permission of the American Chemical Society.

Based on all results discussed above, the ssiRNAs A_n_/T_n_, A_n_/A_n_, and T_n_/T_n_ (*n* = 5 or 7) were selected for further in vitro experiments. Taking again the Hsp27 as the target gene in different cancer cell lines, the efficiency and specificity of the gene silencing effect were evaluated both at the mRNA and protein levels [57]. Figure 16 illustrates some of the results obtained in these tests.

As seen in this figure, the G_5_ TEA-core dendrimer/non-sticky siRNA (A_2_/T_2_) assembly confirmed its inability to elicit gene silencing in all cell lines, while, in agreement with computational predictions, a substantial effect (approximately 90%) was achieved with ssiRNAs bearing complementary overhangs (i.e., A_n_/T_n_). Moreover, again in line with *in silico* results, ssiRNAs ending with non-complementary sequences can induce up to 70% gene silencing, as also seen in Figure 16. Specifically, for these systems, as revealed by simulations, if, on the one side, a higher overhang rigidity leads to stronger nanovector/ssiRNA interaction, on the other side, longer and more flexible overhangs are more beneficial for the subsequent nucleic acid delivery. The results from all *in silico* investigations described above unambiguously supported the conclusion that, among all ssiRNAs with non-complementary overhangs we synthesized to empower low-generation TEA-core PAMAMs with effective delivery capacity, those bearing A_7_/T_7_ terminals represent the best compromise in terms of both nanovector binding and unbinding ability.

**Figure 17:**
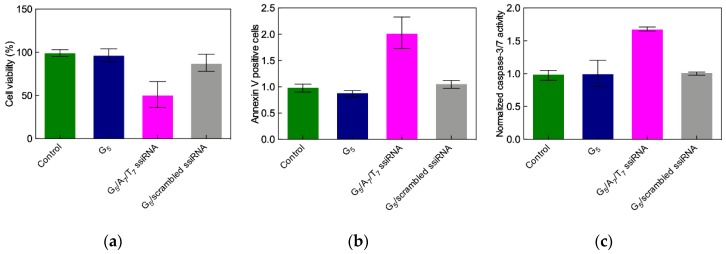
(**a**) Cell proliferation, (**b**) apoptosis, and (**c**) caspase 3/7 activity in PC-3 cells treated with A_7_/T_7_ ssiRNAs (50 nM) delivered by G_5_ TEA-core PAMAM (N/P = 10). Non-treated cells, the G_5_ dendrimer alone and a scrambled (non-silencing) ssiRNA sequence were used for control. Data in panels (**a**) and (**c**) were measured as described in Figure 6. The apoptotic index was measured with fluorescence-activated cell sorting (FACS) flow cytometry by the annexin V assay four days after treatment. Redrawn from [56], with permission of the American Chemical Society.

Before moving the A_7_/T_7_ ssiRNA/G_5_ TEA-core dendrimer complexes to in vivo tests, we further investigated their anticancer effects resulting from Hsp27 silencing in PC-3 cells. Giving the previous results obtained with the delivery of siRNA by the TEA-core dendrimer of Generation 6 and 7 discussed above, we expected this smaller PAMAM to be devoid of toxicity and its complexes with the Hsp27-targeting ssiRNAs to significantly suppress cell growth via a caspase-induced apoptosis. Indeed, as shown in Figure 17a, a notable inhibition of cell proliferation was detected after delivering A_7_/T_7_ ssiRNA with the G_5_ TEA-core nanovector with respect to non-treated cells, cells treated with the G_5_ dendrimer alone, or with a scrambled (non-silencing) ssiRNA.

Concomitantly, fluorescence-activated cell sorting (FACS) flow cytometry revealed a considerable increase of annex V-positive apoptotic cells paralleled with the relevant activation of the apoptotic Caspases 3 and 7. Finally, no toxicity mediated by the ssiRNA/dendrimer complexes was observed via further MTT and lactate dehydrogenase assays, supporting the potential for in vivo experiments with the A_7_/T_7_ ssiRNA/G_5_ TEA-core dendrimer nanoparticles.

#### 3.3.5. In Vivo Delivery of ssiRNAs with G_5_ TEA-Core Dendrimer Nanovectors

**Figure 18:**
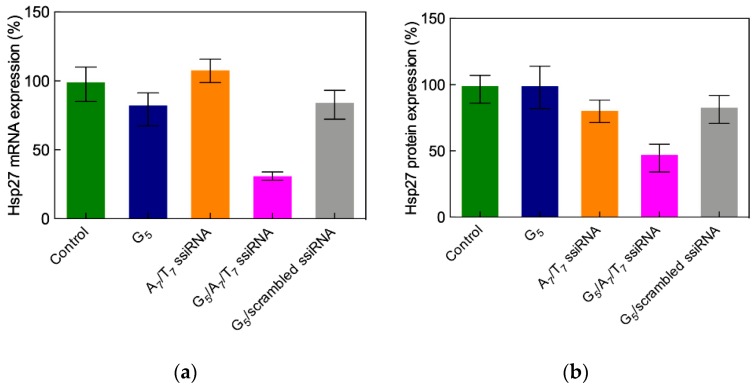

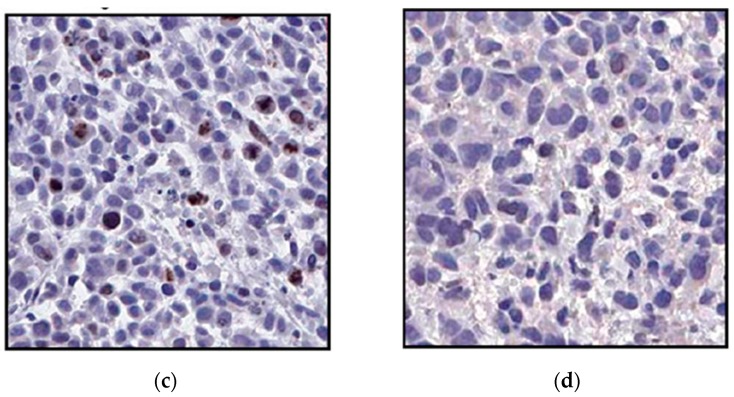
In vivo downregulation of Hsp27 at both mRNA (**a**) and protein (**b**) levels achieved after treating PC-3 cell xenografted nude mice with intratumoral injection of Hsp27 A_7_/T_7_ ssiRNA/G_5_ complex, buffer solution (control), the dendrimer G5 alone, the A7/T7 ssiRNA alone and a scrambled (non-silencing) ssiRNA sequence/G_5_ complex (all used as negative controls). (**c**) Evaluation of tumor cell proliferation via immunohistochemistry using Ki-67 staining after treatment with a scrambled ssiRNA sequence/G_5_ (left) and the Hsp27 A_7_/T_7_ ssiRNA/G_5_ complexes (right). Adapted from [56], with permission of the American Chemical Society.

The final part of the study concerned the evaluation of in vivo gene silencing by the in vitro most efficient ssiRNA/nanovector system. Accordingly, a prostate cancer PC-3 xenografted mouse model was adopted, to which the A_7_/T_7_/G_5_ TEA core dendrimer nanoparticles were slowly administered via slow intratumoral injection. Treatment lasted one week, during which the mice survived well, showing no sign of induced toxicity or weight loss. After mice sacrifice, the expression of Hsp27 in the tumors was measured, as shown in Figure 18a,b. A significant downregulation of Hsp27 at both the mRNA and protein levels was observed, compared to all controls, confirming that the ssiRNA delivered by the dendrimer nanovector was able to elicit potent and specific RNAi also in vivo.

Apoptotic caspase activation was also detected only in mice treated with the nanodelivered ssiRNA, and immunohistochemistry images obtained with Ki-67 antibody staining finally confirmed the remarkable inhibition of cell proliferation in the treated animals (Figure 18c,d).

## 4. Conclusions

In the last ten years, the number of studies involving dendrimers as safe, efficient and effective nanovectors for drug and nucleic acid delivery have increased exponentially. This is mainly due to the exquisite properties of these hyperbranched molecules which, by virtue of their nanoscale size, regularly repeating structure and functional surface groups, make them an ideal drug delivery platform. PAMAM dendrimers in particular bear primary amine groups on their periphery which, being positively charged at physiological pH (7.4), can aptly condense negatively charged nucleic acids for efficient gene or siRNA delivery. In addition, this class of dendrimers features tertiary amines in their interior which become protonated at endosomal pH (5.5), thereby promoting the so-called proton sponge effect and the subsequent release of their DNA/siRNA cargo in the cell cytoplasm.

During the same decade, our group has been particularly active in the field of design and optimization of PAMAM-based dendrimers for siRNA delivery. In particular, we designed, synthesized, and tested highly flexible triethanolamine-core PAMAM dendrimers which proved to be highly effective for siRNA delivery in cancer therapeutics both in vitro and in vivo, as discussed in this brief review. Based on its successful performance, the G_5_ TEA-core PAMAM dendrimer was scheduled to enter clinical trials for siRNA-based cancer therapy in 2014; unfortunately, however, due to the unavailability of GMP dendrimer material, the foreseen clinical trial was delayed and ultimately replaced by the use of Smarticles^®^ for the delivery of siRNA therapeutics [59].

Since the GPM production of dendrimer is quite a challenging process, we decided to exploit the quintessence of nanotechnology, i.e., the controlled self-assembly of small, synthetically amenable building blocks to generate nanosystems for siRNA delivery. Accordingly, we designed, synthesized, and tested amphiphilic dendrons which, upon auto-organization into micelles, were able to mimic the covalent, high generation dendrimers in size, structure and function—in particular for siRNA delivery. These exciting self-assembled nanovectors will be the subject of the second part of this review work.

## Figures and Tables

**Table 1:** *In silico* normalized free energy of binding (ΔG_bind_/*N*) and its major components (binding enthalpy ΔH_bind_/*N* and entropy variation –TΔS_bind_/*N*) for G_4_–G_6_ TEA-core and NH_3_-core PAMAMs in complex with heat shock protein 27 (Hsp27) small interfering RNA (siRNA) at pH 7.4 and 0.15 M NaCl. The normalization factor *N* is the generation-specific total number of charged dendrimer terminal groups (*N*). All values are expressed in kcal/mol^1^. Adapted from [35] with permission of John Wiley and Sons.

	TEA-Core PAMAMs			NH_3_-Core PAMAMs		
G	ΔG_bind_/N	ΔH_bind_/N	−TΔS_bind_/N	ΔG_bind_/N	ΔH_bind_/N	−TΔS_bind_/N
4	−7.57 ^1^	−9.82	2.25	−4.57	−8.02	3.45
5	−14.9	−17.9	3.02	−11.5	−16.0	4.43
6	−17.0	−20.5	3.55	−14.1	−18.8	4.77

^1^ Standard deviation for all data in Table 1 is less that 1%.

## Supplementary Materials

The following are available online at https://www.mdpi.com/1999-4923/11/7/351/s1, Table S1, Computational details.

## Appendix A

**Table A1:** Total and effective enthalpy (ΔH_bind_, ΔH_bind,eff_), entropy (-TΔS_bind_, -TΔS_bind,eff_), and free energy of binding (ΔG_bind_ = ΔH_bind_−TΔS_bind_, ΔG_bind,eff_ = ΔH_bind,eff_−TΔS_bind,eff_) for ssiRNAs featuring complementary and non-complementary overhangs of different length with G_5_ TEA-core PAMAM dendrimers. All values are in kcal/mol (standard deviation for all data in Table A1 is less that 3%). N_eff_ is the number of positive dendrimer charges effectively involved in ssiRNA binding. Adapted from [57] with permission of the American Society of Chemistry.

Overhangs	ΔH_bind_	−TΔS_bind_	ΔG_bind_	N_eff_	ΔH_bind,eff_	−TΔS_bind,eff_	ΔG_bind,eff_
T_2_/T_2_	−571.1	254.3	−316.8	38	−415.6	190.7	−224.9
T_5_/T_5_	−609.9	265.1	−344.8	41	−503.1	241.1	−262.0
A_5_/A_5_	−659.7	249.8	−409.9	46	−592.0	227.2	−364.8
A_5_/T_5_	−637.4	250.0	−387.4	44	−554.9	233.4	−321.5
T_7_/T_7_	−678.4	276.2	−402.2	45	−626.7	258.9	−367.8
A_7_/A_7_	−714.8	266.9	−447.9	52	−669.1	243.6	−425.5
A_7_/T_7_	−690.2	267.3	−422.9	47	−637.3	248.2	−389.1

**Table A2:** Total and effective enthalpy (ΔH_bind_, ΔH_bind,eff_), entropy (−TΔS_bind_, −TΔS_bind,eff_), and free energy of binding (ΔG_bind_ = ΔH_bind_−TΔS_bind_, ΔG_bind,eff_ = ΔH_bind,eff_−TΔS_bind,eff_) for the two dimeric ssiRNAs with G_5_ TEA-core PAMAM dendrimers. All values are in kcal/mol (standard deviation for all data in Table A2 is less that 3%). N_eff_ is the number of positive dendrimer charges effectively involve in ssiRNA binding. Adapted from [57] with permission of the American Society of Chemistry.

Overhangs	ΔH_bind_	−TΔS_bind_	ΔG_bind_		ΔH_bind,eff_	−TΔS_bind,eff_	ΔG_bind,eff_
(A_5_/A_5_)_2_	−1382.7	407.3	−975.4	96	−1260.3	372.7	−887.6
(A_7_/T_7_)_2_	−1480.4	455.9	−1024.5	107	−1441.2	437.2	−1004.0

**Table A3:** Synergistic effect of the dimeric ssiRNA concatemerization on their G_5_ TEA-core PAMAM dendrimer effective binding thermodynamics with respect to the corresponding monomeric ssiRNAs. Data from Table A1 and Table A2. All values are in kcal/mol.

	(A_5_/T_5_)_2_	2 × (A_5_/T_5_)	(A_7_/T_7_)_2_	2 × (A_7_/T_7_)
N_eff_	96	88	107	94
ΔH_bind,eff_	−1260.3	−1109.8	−1441.2	−1274.6
−TΔS_bind,eff_	372.7	446.8	437.2	496.4
ΔG_bind,eff_	−887.6	−663.0	−1004.0	−778.2
