# Supplementary material for: Evolution from Covalent to Self-Assembled PAMAM-Based Dendrimers as Nanovectors for siRNA Delivery in Cancer by Coupled In Silico-Experimental Studies. Part I: Covalent siRNA Nanocarriers
Source: Pharmaceutics. 2019 Jul 18;11(7):351. doi: 10.3390/pharmaceutics11070351 (PMC6680565; doi:10.3390/pharmaceutics11070351)
Supplement: Supplementary file 1 [file pharmaceutics-11-00351-s001.pdf]

pharmaceutics

Supplementary Materials: Evolution from Covalent to Self-Assembled PAMAM-Based Dendrimers as Nanovectors for siRNA Delivery in Cancer by Coupled In Silico-Experimental Studies. Part I: Covalent siRNA Nanocarriers

Domenico Marson, Erik Laurini *, Suzana Aulic, Maurizio Fermeglia and Sabrina Pricl

Table 1. Selected examples of other poly(amidoamine) (PAMAM) dendrimer-based applications in small interfering RNA (siRNA) delivery.

PAMAM Generation (G_n)	Cancer Target of Delivered siRNA	Reference
G_1	E6 and E7 oncogenes	[1]
G_2	E6 and E7 oncogenes	[1]
G_3	E6 and E7 oncogenes	[1]
G_4	E6 and E7 oncogenes	[1]
G_4	Human ether-à-go-go-related gene	[2]
G_4	Vascular endothelial growth factor A	[3]
G_4	Polo-like kinase 1	[4]
G_4	B-cell lymphoma 2	[5]
G_4	Epidermal growth factor receptor	[6]
G_4	B-cell lymphoma 2	[7]
G_5	Astrocyte elevated gene-1	[8]
G_5	B-cell lymphoma 2	[9]
G_5	Multidrug resistant protein 1	[10]
G_5	Polo-like kinase 1	[11]
G_5	Major vault protein	[12]
G_6	Carbonyl reductase 1	[13]

Determination of the Binding Free Energy of the TEA-Core Dendrimers and the Different siRNAs

To estimate the free energy of binding ΔG_{bind} between triethanolamine (TEA)-core poly(amidoamine) (PAMAM) dendrimers and all small interfering RNA (siRNA) molecules, including the two concatemers, we resorted to a well-established computational recipe based on the so-called Molecular Mechanics/Poisson–Boltzmann Surface Area (MM/PBSA) methodology [14–19]. Briefly, for a non-covalent association of two molecular entities $A + B \geq AB$, the free energy of binding involved in the process may be generally written as $\Delta G_{bind} = G_{AB} - G_A - G_B$.

For any species on the right hand side of this equation, from basic thermodynamics we have $G_i = H_i - TS_i$, where H_i and S_i are the enthalpy and entropy of the i-th species, respectively, and T is the absolute temperature. In view of this expression, ΔG_{bind} can then be written as: $\Delta G_{bind} = \Delta H_{bind} - T\Delta S_{bind}$.

ΔH_{bind} is the variation in enthalpy upon association and, in the MM/PBSA framework of the theory, can be calculated by summing the molecular mechanics energies (ΔE_{MM}) and the solvation free energy (ΔG_{solv}), i.e., $\Delta H_{bind} = \Delta E_{MM} + \Delta G_{solv}$.

ΔH_{bind} is obtained directly from a single molecular dynamics (MD) trajectory of the molecular complex as $\Delta E_{MM} = \Delta E_{vdW} + \Delta E_{ele}$, where ΔE_{vdW} is the variation of the nonbonded van der Waals energy and ΔE_{ele} is the electrostatic contribution calculated from the Coulomb potential. The solvation/desolvation contribution to the free energy, ΔG_{solv}, can also be also split in two components: $\Delta G_{solv} = \Delta G_{PB} + \Delta G_{NP}$.

The calculations of the polar solvation term ΔG_{PB} were done with the DelPhi package [20], with interior and exterior dielectric constants equal to 1 and 80, respectively. The dielectric boundary is the contact surface between the radii of the solute and the radius (1.4 Å) of a water molecule. The

linearized Poisson–Boltzmann equations were solved on a cubic lattice by using the iterative finite-difference method implemented in DelPhi. A grid spacing of 2/Å, extending 20% beyond the dimensions of the solute, was employed. Potentials at the boundaries of the finite-difference lattice were set to the sum of the Debye–Hückel potentials. The non-polar component ΔG_{NP} was obtained using the following relationship [21]: $\Delta G_{NP} = \gamma$ SA + β, in which γ = 0.00542 kcal/(mol Å^2), β = 0.92 kcal/mol, and the molecular surface area SA was estimated by means of the MSMS software [22].

The quasi-harmonic analysis approach [23] was followed to estimate the last parameter, i.e., the change in solute entropy upon association $-T\Delta S_{bind}$. On the basis of MD snapshots of the complex, we generated structures of the uncomplexed reactants by removing the atoms of the dendrimer and siRNA, respectively. Each of those structures was minimized using a distance-dependent dielectric constant ε = 4r to account for solvent screening, and its entropy was calculated using classical statistical formulas and normal mode-analysis. To minimize the effects due to different conformations adopted by individual snapshots, we averaged the estimation of entropy over 200 snapshots.

Finally, the effective number of charges involved in binding, and the corresponding effective free energy of binding values (Tables A1–A3) were obtained by performing a per residue binding free energy decomposition exploiting the MD trajectory of each given siRNA/dendrimer complex. This analysis was carried out using the MM/GBSA approach [24] and was based on the same snapshots used in the binding free energy calculation.

Steered Molecular Dynamics (SMD) of G_5/siRNA Unbinding

The purpose of a MD-based study of the dissociation of a siRNA molecule from its dendrimer complex is the determination of the transition from a bound state (BS) to an unbound state (UBS) along a pathway coordinate $\xi(\mathbf{r})$ (e.g., the distance between the dendrimer and the nucleic acid fragment). This pathway is typically characterized by a substantially high free energy barrier, i.e., several times k_bT. Under these circumstances, the probability to observe a spontaneous barrier-crossing event within a reasonable simulation time is extremely low. Therefore, in order to supersede this obstacle, a time-dependent external potential energy function U can be applied to the system under study to drive the reaction from BS to UBS. If the pathway coordinate $\xi(\mathbf{r})$ is a function of atomic positions, then the external potential energy function $U(\xi(\mathbf{r}),t)$ is called a steering function. $U(\xi(\mathbf{r}),t)$ is generally chosen harmonic and centered on a given reference reaction coordinate $\xi_0(t)$ so that: $U(\xi(\mathbf{r}),t) = (k_{CM}/2) \times (\xi(\mathbf{r}) - \xi_0(t))^2$.

The reference reaction coordinate 0(t) is changed typically with a constant velocity so that: $\xi_0(t) = \xi_{BS} + v_\xi t$.

The harmonic constant k_{CM} and the steering velocity v_ξ are then the two parameters of a steered molecular dynamics (SMD) simulation. In any SMD experiment, both the steering function $\xi_0(t)$ and the atoms to which it must be applied should be chosen very carefully. For big molecules with rather loose structures which are bound by strong interactions such as the G_5 TEA-core dendrimer and a siRNA complex, the classical approaches of i) applying a pulling force on a single atom or ii) defining ξ as the distance between the two centers of mass of the two molecules in the complex are not appropriate. In fact, in this case a single point force (or mass weighted uniform force) will induce the stretching of the nucleic acid with unrealistic structure distortion and a partial unwinding before the unbinding process takes place.

An additional problem might arise during conventional SMD unbinding simulations of a big supermolecular systems such as G_5/siRNA if the interaction between the dendrimer and the nucleic acid is spread over a large surface perpendicular to the pulling direction. If, during the pulling phase, one side of the interface unbinds while the other remains bounded, a force moment is generated with respect to the center of mass. This results in a rotation of the two macromolecules, which roll on each other instead of falling apart.

To avoid both stretching and rolling artifacts, we applied a procedure based on the so-called individual pulling method, recently proposed by Cuendet and Michielin [25]. Briefly, the reference position of an atom in an MD equilibrated structure of a given G_5/siRNA complex is determined with

respect to the center of mass of its respective unit (dendrimer or siRNA). A harmonic potential energy term centered on this reference point is then applied to the z-coordinate of the atom in a xyz coordinate reference system, while movement in the lateral plane is left free. This corresponds to a restraint of the root-mean-square deviation (RMSD) along the z-axis in each molecular unit of the complex. To realize pulling, the reference position of all restrained atoms is shifted uniformly along the z-axis—each unit (nanocarrier and cargo) in opposite directions. During this process, the reference structures remain unchanged in each part, while the distance between the centers of mass is increased. The physical and mathematical concepts underlying the individual pulling method in SMD are given in full in reference [12].

For the SMD simulation setup, decorrelated configurations with the velocities of each G_5/siRNA complex system were chosen from the corresponding equilibrated MD trajectories as starting points for the pulling runs. In detail, 180 snapshots taken from the last 90 ns of each MM/PBSA MD production run were employed to calculate the average center of mass distance ξ_{BS}, as well as the average internal coordinates. This initial structural was used as initial distance and reference structure for the individual restraints. Next, these restraints were applied on the atoms specified above, with a resulting harmonic constant k_{CM} equal to 2×10^4 kJ/(mol nm^2), a value stiff enough to have a good spatial resolution in the free energy profile while not damping the thermal behavior of the system. The steered system was then allowed to further equilibrate for 0.5 ns with 0 fixed to ξ_{BS}. Finally, the pulling itself was started. A pulling speed ν of 5×10^{-4} nm/ps was chosen as a trade-off between staying as close to equilibrium as possible and keeping the computing time within manageable limits. With these settings, 2 nm were covered in 4 ns of the SMD simulation.

References

1. Dutta, T.; Burgess, M.; McMillan, N.A.J.; Parekh, H.S. Dendrosome-based delivery of siRNA against E6 and E7 oncogenes in cervical cancer. *Nanomedicine* **2010**, *6*, 463–470.
2. Li, G.; Hu, Z.; Yin, H.; Zhang, Y.; Huang, X.; Wang, S.; Li, W. A novel dendritic nanocarrier of polyamidoamine-polyethylene glycol-cyclic RGD for "smart" small interfering RNA delivery and in vitro antitumor effects by human ether-à-go-go-related gene silencing in anaplastic thyroid carcinoma cells. *Int. J Nanomed.* **2013**, *8*, 1293–1306.
3. Xu, L.; Yeudall, W.; Yang, H. Folic acid-decorated polyamidoamine dendrimer exhibits high tumor uptake and sustained highly localized retention in solid tumors: Its utility for local siRNA delivery. *Acta Biomater.* **2017**, *57*, 251–261.
4. Ohyam, A.; Higashi, T.; Motoyama, K.; Arima, H. Ternary complexes of folate-PEG-appended dendrimer (G4)/α-cyclodextrin conjugate, siRNA and low-molecular-weight polysaccharide sacran as a novel tumor-selective siRNA delivery system. *Int. J. Biol. Macromol.* **2017**, *99*, 21–28.
5. Patil, M.L.; Zhang, M.; Minko, T. Multifunctional triblock nanocarrier (PAMAM-PEG-PLL) for the efficient intracellular siRNA delivery and gene silencing. *ACS Nano* **2011**, *53*, 1877-1887.
6. Agrawal, A.; Min, D.H.; Singh. N.; Zhu, H.; Birjiniuk, A.; von Maltzahn, G.; Harris, T.J.; Xing, D.; Woolfenden, S.D.; Sharp, P.A.; Charest, A.; Bhatia, S. Functional delivery of siRNA in mice using dendriworms. *ACS Nano* **2009**, *39*, 2495–2504.
7. Felber, A.E.; Castagner, B.; Elsabahy, M.; Deleavey, G.F.; Damha, M.J.; Leroux, J.C. siRNA nanocarriers based on methacrylic acid copolymers. *J. Control. Release* **2011**, *152*, 159–167.
8. Rajasekaran, D.; Srivastava, J.; Ebeid, K.; Gredler, R.; Akiel, M.; Jariwala, N.; Robertson, C.L.; Shen, X.N.; Siddiq, A.; Fisher, P.B.; Salem, A.K.; Sarkar, D. Combination of nanoparticle-delivered siRNA for astrocyte elevated gene-1 (AEG-1) and all-trans retinoic acid (ATRA): an effective therapeutic strategy for hepatocellular carcinoma (HCC). *Bioconjug. Chem.* **2015**, *26*, 1651–1661.
9. Wu, D.; Yang, J.; Xing, Z.; Han, H.; Wang, T.; Zhang, A.; Yang, Y.; Li, Q. Phenylboronic acid-functionalized polyamidoamine-mediated Bcl-2 siRNA delivery for inhibiting the cell proliferation. *Colloids Surf. B Biointerfaces* **2016**, *146*, 318–325.
10. Li, J.; Liu, J.; Guo, N.; Zhang, X. Reversal of multidrug resistance in breast cancer MCF-7/ADR cells by h-R3-siMDR1-PAMAM complexes. *Int. J. Pharm.* **2016**, *511*, 436–445.

11. Li, J.; Liu, J.; Li, S.; Hao, Y.; Chen, L.; Zhang, X. Antibody h-R3-dendrimer mediated siRNA has excellent endosomal escape and tumor targeted delivery ability, and represents efficient siPLK1 silencing and inhibition of cell proliferation, migration and invasion. *Oncotarget* **2016**, *7*, 13782–13796.
12. Han, M.; Lv, Q. Tang, X.J.; Hu, Y.L.; Xu, D.H.; Li, F.Z.; Liang, W.Q.; Gao, J.Q. Overcoming drug resistance of MCF-7/ADR cells by altering intracellular distribution of doxorubicin via MVP knockdown with a novel siRNA polyamidoamine-hyaluronic acid complex. *J. Control. Release* **2012**, *163*, 136–144.
13. Kobayashi, A.; Yokoyama, Y.; Osawa, Y.; Miura, R.; Mizunuma, H. Gene therapy for ovarian cancer using carbonyl reductase 1 DNA with a polyamidoamine dendrimer in mouse models. *Cancer Gene Ther*. **2016**, *23*, 24–28.
14. Karatasos, K.; Posocco, P.; Laurini, E.; Pricl, S. Poly(amidoamine)-based dendrimer/siRNA complexation studied by computer simulations: effects of pH and generation on dendrimer structure and siRNA binding. *Macromol Biosci*. **2012**, *12*, 225–240.
15. Posocco, P.; Laurini, E.; Dal Col, V.; Marson, D.; Karatasos, K.; Fermeglia, M., Pricl, S. Tell me something that I do not know. Multiscale molecular modeling of dendrimer/dendron organization and self-assembly in gene therapy. *Curr. Med. Chem*. **2012**, *19*, 5062–5087.
16. Posocco, P.; Laurini, E.; Dal Col, V.; Marson, D.; Peng, L.; Smith, D.K.; Klajnert, B.; Bryszewska, M.; Caminade, A.-M.; Majoral, J.P.; Fermeglia, M.; Karatasos, K.; Pricl, S. Multiscale modeling of dendrimers and dendrons for drug and nucleic acid delivery. In *Dendrimers in Biomedical Applications*; Klajnert, B., Peng, L., Ceña, V., Eds.; RSC Publishing: Cambrige, United Kingdom, 2013; pp. 148–166.
17. Pavan, G.M.; Posocco, P.; Tagliabue, A.; Maly, M.; Malek, A.; Danani, A.; Ragg, E.; Catapano, C.V., Pricl, S. PAMAM dendrimers for siRNA delivery: computational and experimental insights. *Chem. Eur. J.* **2010**, *16*, 7781–7795.
18. Marson, D.; Laurini, E.; Posocco, P.; Fermeglia, M.; Pricl, S. Cationic carbosilane dendrimers and oligonucleotide binding: an energetic affair. *Nanoscale* **2015**, *7*, 3876–3887.
19. Mehrabadi, F.S.; Hirsch, O.; Zeisig, R.; Posocco, P.; Laurini, E.; Pricl, S.; Haag, R.; Kemmner, W.; Calderón, M. Structure–activity relationship study of dendritic polyglycerolamines for efficient siRNA transfection. *RSC Adv*. **2015**, *5*, 78760–78770.
20. Gilson, M.K.; Sharp, K.A.; Honig, B. Calculating the electrostatic potential of molecules in solution: method and error assessment. *J. Comput. Chem*. **1988**, *9*, 327–335.
21. Sitkoff, D.; Sharp, K. A.; Honing, B. Accurate calculation of hydration free energies using macroscopic solvent models. *J. Phys. Chem*. **1994**, *98*, 1978–1988.
22. Sanner, M. F.; Olson, A. J.; Spehner, J.-C. Reduced surface: an efficient way to compute molecular surfaces. *Biopolymers* **1996**, *38*, 305–320.
23. Andricioaei, I.; Karplus, M. J. On the calculation of entropy from covariance matrices of the atomic fluctuations. *J. Chem, Phys.* **2001**, *115*, 6289–6292.
24. Tsui, V.; Case, D. A. Theory and applications of the generalized Born solvation model in macromolecular simulations. *Biopolymers* **2000**, *56*, 275–291.
25. Cuendet, M. A.; Michielin, O. Protein-protein interaction investigated by steered molecular dynamics: the TCR-pMHC complex. *Biophys. J.* **2008**, *95*, 3575–3590.

© 2019 by the authors. Submitted for possible open access publication under the terms and conditions of the Creative Commons Attribution (CC BY) license (http://creativecommons.org/licenses/by/4.0/).
